# Cell cycle-dependent activity of the novel dual PI3K-MTORC1/2 inhibitor NVP-BGT226 in acute leukemia

**DOI:** 10.1186/1476-4598-12-46

**Published:** 2013-05-24

**Authors:** Kerstin Maria Kampa-Schittenhelm, Michael Charles Heinrich, Figen Akmut, Katharina Henriette Rasp, Barbara Illing, Hartmut Döhner, Konstanze Döhner, Marcus Matthias Schittenhelm

**Affiliations:** 1 Department of Hematology, Oncology, Rheumatology, Immunology and Pulmology, University Hospital Tübingen, Otfried-Müller-Straße 10, 72076, Tübingen, Germany; 2 Portland VA Medical Center and OHSU Knight Cancer Institute, Portland, OR, USA; 3 Department of Internal Medicine III, University Hospital Ulm, Ulm, Germany

**Keywords:** Leukemia, PI3K, AKT, NVP-BEZ235, NVP-BGT226

## Abstract

**Background:**

Dysregulation of the PI3Kinase/AKT pathway is involved in the pathogenesis of many human malignancies. In acute leukemia, the AKT pathway is frequently activated, however mutations in the PI3K/AKT pathway are uncommon. In some cases, constitutive AKT activation can be linked to gain-of-function tyrosine kinase (TK) mutations upstream of the PI3K/AKT pathway. Inhibitors of the PI3K/AKT pathway are attractive candidates for cancer drug development, but so far clinical efficacy of PI3K inhibitors against various neoplasms has been moderate. Furthermore, specific MTORC1 inhibitors, acting downstream of AKT, have the disadvantage of activating AKT via feed-back mechanisms. We now evaluated the antitumor efficacy of NVP-BGT226, a novel dual pan-PI3K and MTORC1/2 inhibitor, in acute leukemia.

**Methods:**

Native leukemia blasts were stained to analyze for AKT phosphorylation levels on a flow cytometer. Efficacy of NVP-BGT226 in comparison to a second dual inhibitor, NVP-BEZ235, was determined with regard to cellular proliferation, autophagy, cell cycle regulation and induction of apoptosis in *in vitro* and *ex vivo* cellular assays as well as on the protein level. An isogenic AKT-autoactivated Ba/F3 model, different human leukemia cell lines as well as native leukemia patient blasts were studied. Isobologram analyses were set up to calculate for (super) additive or antagonistic effects of two agents.

**Results:**

We show, that phosphorylation of AKT is frequently augmented in acute leukemia. NVP-BGT226 as well as NVP-BEZ235 profoundly and globally suppress AKT signaling pathways, which translates into potent antiproliferative effects. Furthermore, NVP-BGT226 has potent proapoptotic effects *in vitro* as well as in *ex vivo* native blasts. Surprisingly and in contrast, NVP-BEZ235 leads to a profound G1/G0 arrest preventing significant induction of apoptosis. Combination with TK inhibitors, which are currently been tested in the treatment of acute leukemia subtypes, overcomes cell cycle arrest and results in (super)additive proapoptotic effects for NVP-BGT226 – but also for NVP-BEZ235. Importantly, mononuclear donor cells show lower phospho-AKT expression levels and consequently, relative insensitivity towards dual PI3K-MTORC1/2 inhibition.

**Conclusions:**

Our data suggest a favorable antileukemic profile for NVP-BGT226 compared to NVP-BEZ235 – which provides a strong rationale for clinical evaluation of the dual PI3K-MTORC1/2 inhibitor NVP-BGT226 in acute leukemia.

## Background

Most subtypes of acute leukemia remain difficult to treat. Patients typically respond to initial induction treatment regimens - but the majority of adult patients relapse and die of their disease. Novel therapeutic strategies include molecular targeted therapeutics, such as tyrosine kinase inhibitors (TKI) targeting wildtype and gain-of-function mutated isoforms of the *FLT3*, *KIT* and *ABL1* tyrosine kinases [1,2]. However, clinical benefit of these agents is typically restricted to distinct subsets of patients and/or is minimal to moderate [3-7].

The phosphoinositide 3-kinase (PI3K)/AKT pathway is a critical regulator of cellular viability, including insulin metabolism, protein synthesis, proliferation, and apoptosis [8]. Dysregulation of the PI3K kinase/AKT pathway is involved in pathogenesis of many human malignancies - including leukemia [9-12]. In many types of solid tumors, activated AKT signaling can be linked to distinct gene mutations promoting constitutive AKT activation (e.g. PIK3CA [13] or AKT [14] mutations) or preventing attenuation of the AKT signal transduction pathway (PTEN [15,16] mutations). While, these mutations are rare in acute leukemias [17,18] constitutive phosphorylation of AKT is nevertheless frequently found. In some cases, activation of AKT can be linked to gain-of-function tyrosine kinase mutations [19]. However, in most cases of acute leukemia with detectable activation of the PI3K/AKT pathway, the molecular mechanisms are unknown.

Targeting the PI3K/AKT pathway is an attractive therapeutic strategy and various small molecule inhibitors are under clinical investigation [20]. Proof of principle for the clinical potential to inhibit the PI3K/AKT pathway in human neoplasms was provided by the successful development of rapamycin-derivatives in the treatment of advanced renal cell carcinoma (RCC), where temsirolimus provides a significant overall survival benefit [21]. Rapamycin and its analogues are highly specific inhibitors of the serine/threonine mammalian target of rapamycin kinase (mTOR). Although an antileukemic activity of rapamycin has been reported in some patients with AML [22] it is now believed that several resistance mechanisms may prevent activity of rapamycin therapy in leukemia: Two mTOR complexes have been described, of which only the raptor (regulatory associated protein of mTOR) associated MTOR-complex 1 (a downstream regulator of AKT signaling) is a target of rapamycin - whereas the rictor (rapamycin-insensitive companion of mTOR)-regulated MTOR complex 2 (a crucial activator of AKT via serine-phosphorylation at codon 473) is not affected by rapamycin inhibition. Even more, MTORC1 inhibition results in increased PI3K/AKT but also MAPK activity via strong negative feedback loop mechanisms [23-26]. Consequently, specific inhibitors globally and sustainably suppressing PI3K/AKT signaling pathways may provide an improved antitumor response.

We herein provide evidence that AKT is frequently phosphorylated and exclusively augmented in native leukemia samples compared to physiologic mononuclear cells, making the PI3K/AKT pathway an attractive target in the treatment of acute leukemia.

In an attempt to globally block PI3K/AKT/MTORC signaling we tested the antileukemic potency of a novel pan class I PI3K and MTORC1 plus MTORC2 inhibitor, NVP-BGT226 [27], in comparison to a second dual inhibitor (NVP-BEZ235 [28]) currently widely under clinical investigation – including acute leukemia (European Clinical Trials Database number EUDRACT2011-005050-61).

Our data will provide a strong rationale for clinical evaluation of NVP-BGT226 in acute leukemias with activated PI3K/AKT signaling.

## Results

### AKT is maximally activated in acute leukemia

The PI3K-AKT signal transduction pathway is frequently activated in acute leukemias (recently reviewed by Polak and Buitenhuis [29]). Moreover, mice transplanted with AKT-activated hematopoietic stem cells develop acute leukemia, indicating the leukemogenic potential of an activated PI3K/AKT pathway [9].

Maximal activation of AKT results from the phosphorylation of threonine and serine residues at positions 308 (Thr) and 473 (Ser). We addressed whether AKT is activated in acute leukemia and evaluated phospho-AKT expression levels of native acute leukemia blood and/or bone marrow samples (total n=62) collected from adult patients with newly diagnosed AML or mixed phenotype and lymphoblastic leukemia.

**Figure 1:**
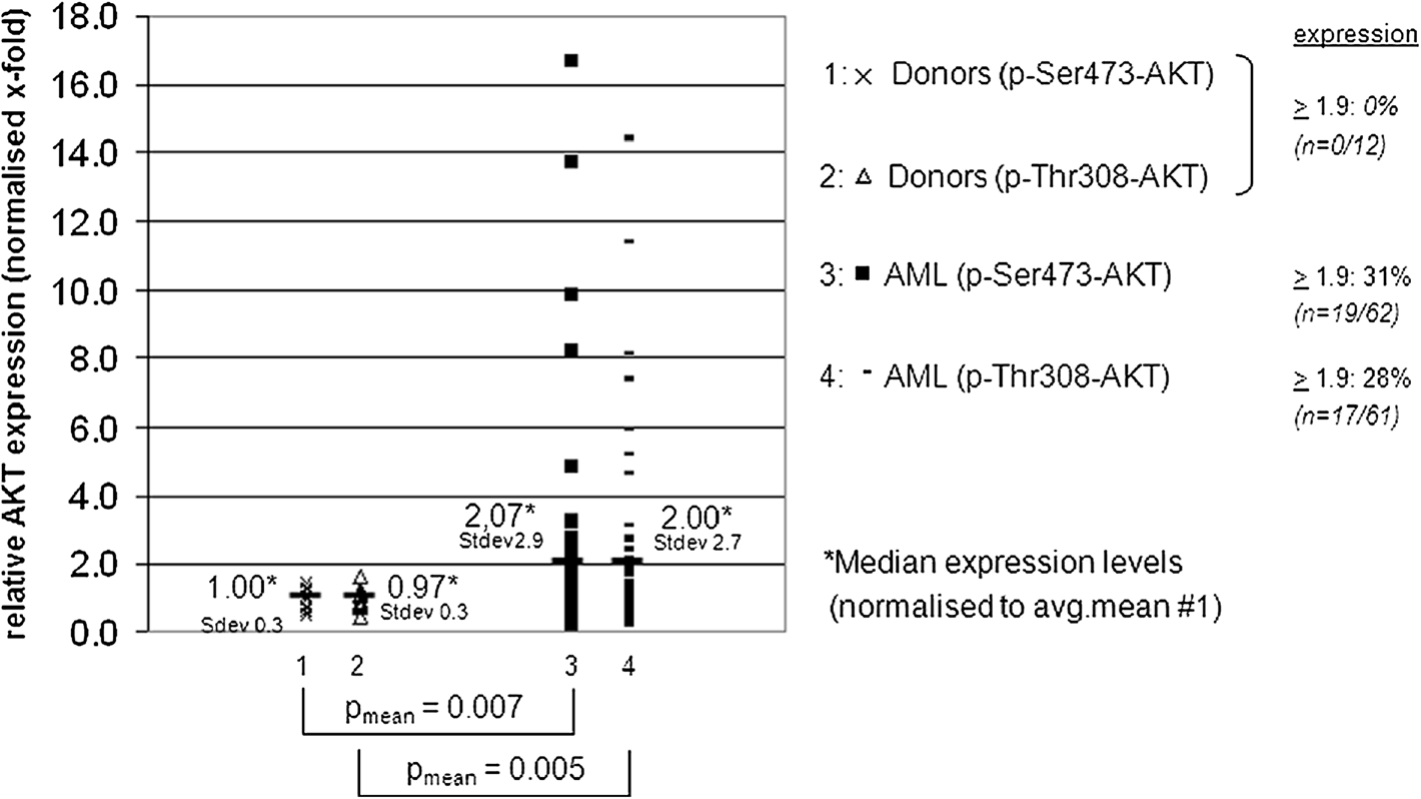
**Assessment of Thr308/Ser473 phosphorylation of AKT in native leukemia cells.** Intracellular expression levels of 12 blood and bone marrow donor samples and 62 leukemia patient samples were assessed flow cytometrically. Relative expression analyses compared to an unspecific IgG control reveal frequent global AKT phosphorylation in hematopoietic benign and malignant mononuclear cells - whereas mean phosphorylation levels of both Ser473 and Thr308 are statistically significantly increased in leukemia. Augmented phospho-AKT expression is exclusive to leukemia specimens. Patient characteristics are provided with Additional file 1: Table S1.

A flow cytometry-based intracellular immunostain was set up to assay for Thr308 and Ser473 phosphorylation patterns in native leukemia blasts. In addition, phospho-AKT expression levels of physiologic hematopoietic blasts derived from healthy blood and bone marrow donors (n=12) were determined. Relative ratios compared to unspecific IgG-staining were calculated and normalized to the median expression level of the healthy donor cohort as shown in Figure 1.

In contrast to the healthy donor cohort, where phospho-AKT expression levels clustered around 1 (1.0 for Ser472 and 0.97 for Thr308) on a normalized relative expression level scale (standard deviation 0.3 each), acute leukemia specimens were frequently found to have augmented phosphorylation patterns of AKT. Phosphorylation levels for both Ser473 as well as Thr308 thereby revealed wide expression variance ranging from sheer absence to ~17-fold increase of phosphorylation levels in leukemia samples compared to the donor cohort. Mean expression levels in the leukemia cohort were statistically significantly higher, with an approximately 2-fold elevation of both Ser473- (p = 0.007) as well as Thr308-phosphorylation (p = 0.005) compared to the healthy donor controls in a student’s t-test.

Notably, strongly phosphorylated specimens were exclusively found in the acute leukemia cohort (≥ two-fold expression above normal was found in ~30% of all tested samples compared to 0% in the donor cohort).

Subanalysis of leukemia blasts derived from bone marrow aspirates (n=23) versus peripheral blood specimens (n=39 (Ser473) or n=38 (Thr308)) revealed no significant difference of phospho-AKT expression at codon Thr308 (p = 0.06) as well as Ser473 (p = 0.09).

Comparative analysis of expression levels with leukemia subclassifications, chromosomal or gene mutation status, leukocyte count, age or gender did not reveal a strong correlation between AKT phosphorylation levels and clincial parameters. This is in contrast to previous reports demonstrating a positive association of Thr308 phosphorylation with high-risk cytogenetics and poor prognosis [30] (patient characteristics are provided with Additional file 1: Table S1 with the online version of the article).

### NVP-BGT226 has antitumor activity in a PTEN-deficient acute leukemia cell line model

Our findings of frequent and augmented phosphorylation of AKT in acute leukemia samples suggest that the AKT pathway is (auto) activated and may provide a promising target for directed therapeutics:

Using Jurkat cells, a PTEN-deficient acute lymphoblastic leukemia cell line rendering AKT signaling pathways autoactivated [31], we now provide evidence that NVP-BGT226 is capable of inhibiting oncogene-driven PI3K/AKT/MTOR signal transduction pathways in acute leukemia.

To better compare efficacy in the context of established compounds, we co-investigated the dual PI3K/MTOR inhibitor NVP-BEZ235. This compound has recently been tested to have significant activity against native leukemia cells [32].

Cell lysates extracted from Jurkat cells treated with NVP-BGT226 or NVP-BEZ235 were immunoblotted together with various phospho-AKT control lysates (treated with the pan-PI3K inhibitor LY294002 or the specific MTORC1 inhibitor rapamycin).

**Figure 2:**
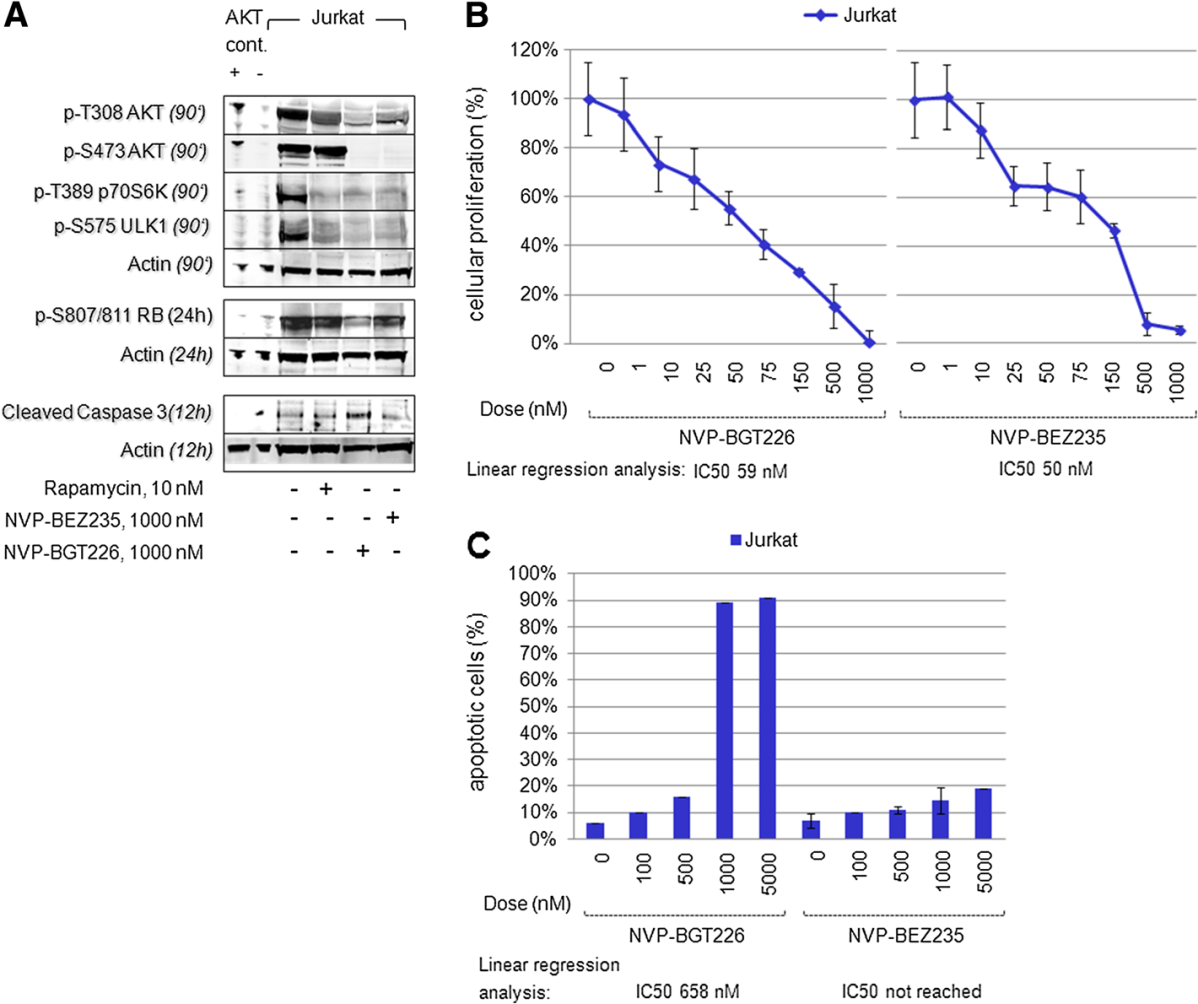
**Dual PI3K/MTOR inhibition is effective in PTEN-deficient AKT-activated acute leukemia cells.** (**A**) Exposure of Jurkat cells to NVP-BGT226, NVP-BEZ235 or Rapamycin reveals preferential consecutive dephosphorylation of AKT at T308 as well as S473 for NVP-BGT226. This results in suppression of downstream targets including p-T389 p70S6K, p-S575 ULK1, p-S807/811 RB and cleavage of caspase 3. (**B**) Dose dilution experiments reveal that NVP-BGT226 as well as NVP-BEZ235 inhibit cellular proliferation in an XTT-based assay. Estimated IC50s, calculated by linear regression dose-effect plots, are provided at the bottom of each graph. (**C**) Assessment of induction of apoptosis shows a preferential proapoptotic effect of NVP-BGT226 when compared to NVP-BEZ235 in an annexin V-based flow cytometry assay. IC50s are provided at the bottom of each graph.

The western blot experiment provided with Figure 2A reveals, that dual inhibition of PI3Kinases and MTOR1/2 complexes by NVP-BGT226 consecutively inhibits serine (S473) as well as threonine (T308) phosphorylation of AKT. Moreover, inhibition of AKT activity leads to potent dephosphorylation of known downstream targets such as p70S6K and retinoblastoma protein (RB) (required for cell growth and G1 cell cycle progression) (reviewed in Panwalkar et al. [33]), ULK1 (a key initiator of MTOR-mediated autophagy when dephosphorylated) [34] and increased cleavage of caspase 3 (a global marker for activated apoptosis cascades).

While similar potency to inhibit S473-AKT and p70S6Kinases was observed for NVP-BGT226 as well as NVP-BEZ235 – the capacity to mediate T308-AKT and RB dephosphorylation as well as cleavage of caspase 3 was more pronounced for NVP-BGT226 compared to NVP-BEZ235.

Suppression of PI3K-AKT-MTORC1/2 signal transduction did translate into a potent antiproliferative effect for both dual PI3K/MTOR inhibitors (Figure 2B) – with similar potency in the lower nanomolar range (IC50s were calculated by linear regression analysis using dose-effect plots).

Surprisingly, a strong discrepancy was noticed for the proapoptotic potential of these two inhibitors. Potent induction of apoptosis was observed for NVP-BGT226, while in contrast, virtually any meaningful proapoptotic effect was measured for NVP-BEZ235 in an annexin V-based assay (Figure 2C). This observation is consistent with immunoblot findings of reduced cleavage intensity of caspase 3 in NVP-BEZ235 treated cells.

### NVP-BGT226 inhibits cellular proliferation and overcomes cell cycle arrest to induce apoptosis in acute leukemia cell lines

**Figure 3:**
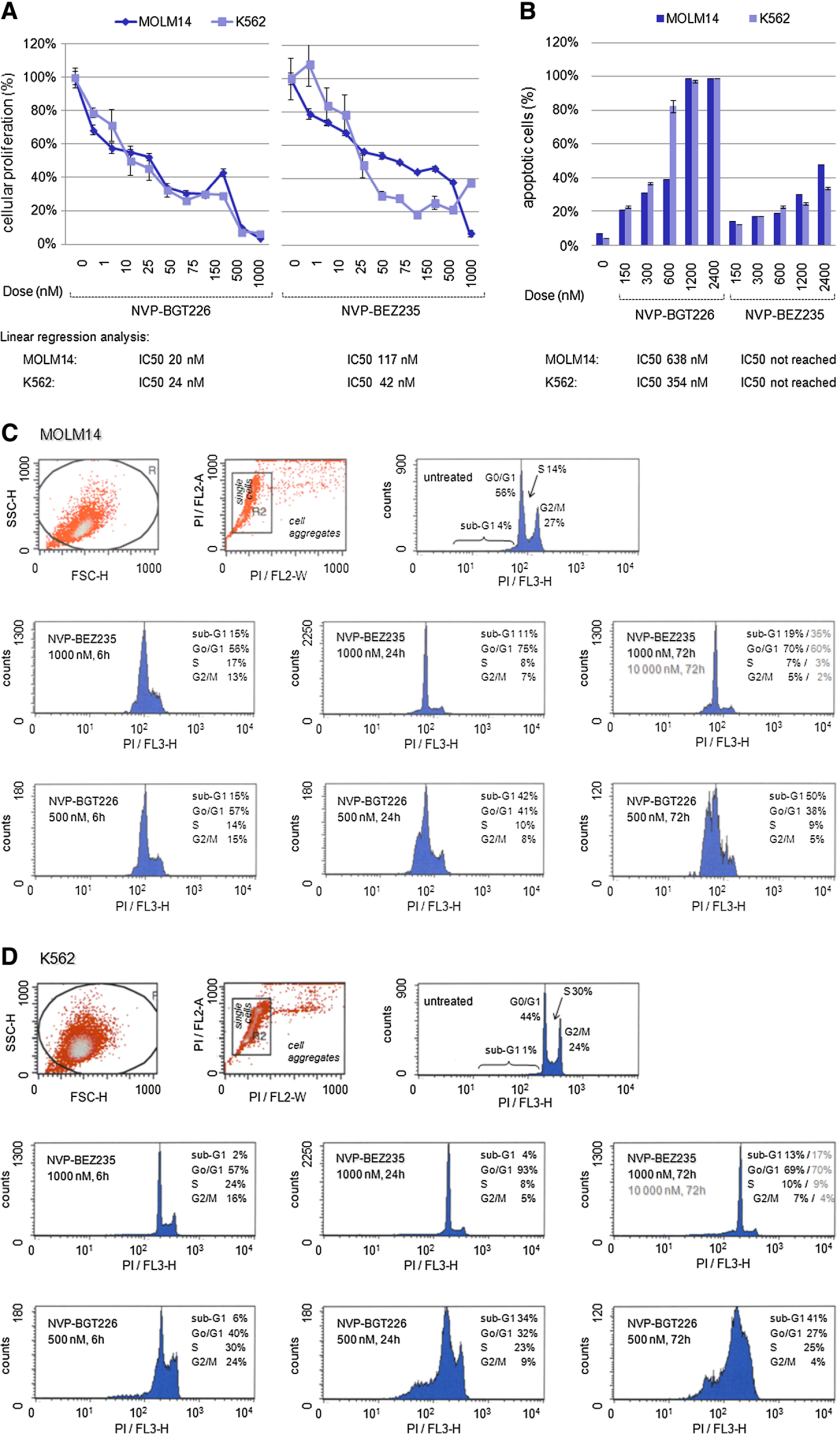
**Evaluation of dual PI3K/MTOR inhibition in mutant-TK AKT-activated acute leukemia cell lines.** (**A**) MOLM14 cells harboring a *FLT3* ITD and K562 cells harboring a *BCR-ABL1* gain-of-function mutation are treated with NVP-BGT226 or NVP-BEZ235 and cellular proliferation is measured using an XTT-based assay. Both inhibitors reveal high antiproliferative potency in both cell lines. IC50s are provided at the bottom of each graph. (**B**) Dual PI3K/MTOR inhibition using NVP-BGT226 or NVP-BEZ235 reveals agent-specific induction of apoptosis in MOLM14 and K562 cells – with NVP-BGT226 the by far more potent agent. Linear regression analysis to calculate IC50s is provided at the bottom of each graph. (**C**) Cell cycle analyses of MOLM14 cells treated with either agent demonstrate strong G1/G0 arrest with failure to induce meaningful apoptosis for NVP-BEZ235 exposed cells. In contrast, NVP-BGT226 treated cells (bottom panels) show a time-dependent increase of the sub-G1/G0 fraction, indicating apoptotic/dead cells. (**D**) Similar effects on cell cycle regulation are shown for the K562 cell line treated with either NVP-BEZ235 or NVP-BGT226 – with a strong G1/G0 arrest for NVP-BEZ235 but potent and time-dependent increase of the apoptotic/dead cell fraction for NVP-BGT226.

To expand our studies to other oncogene-driven AKT-activated leukemia cell models, we chose leukemia cell lines with known gain-of-function tyrosine kinase mutations, which are prevalent in 30-40% of patients with AML (*FLT3* and *KIT)* or ALL (*BCR-ABL1* and *FLT3*) [2]: The acute monocytic leukemia cell line MOLM14 (harboring a *FLT3* ITD mutation) and the CML blast crisis cell line K562 (harboring *a BCR-ABL1* fusion transcript mutation) were exposed to NVP-BGT226 in a dose dependent manner and inhibition of cellular proliferation was determined. In addition, efficacy of NVP-BGT226 was directly compared to NVP-BEZ235. Both inhibitors proved to be highly sensitive with estimated IC50s in the lower nanomolar ranges (<100 nM) for both cell lines (Figure 3A).

When looking at the capacity to induce apoptosis in these leukemia cells, NVP-BGT226 proved to be a strong inducer of programmed cell death in both cell lines. However, estimated IC50s were considerably higher compared to the antiproliferative capacity (Figure 3B).

Interestingly, when treating cells with NVP-BEZ235 only a minor proportion of cells underwent apoptosis with IC50s that were not reached up to doses of 10 000 nM.

The obvious discrepancy of NVP-BGT226 and NVP-BEZ235 to induce apoptosis – while both agents are highly sensitive with regard to inhibition of cellular proliferation, lead us to hypothesize that divergent cell cycle effects may be the reason for this observation.

We treated MOLM14 and K562 cells with ~IC50 doses of NVP-BGT225 (500 nM) or the 2-fold dose of NVP-BEZ235 (1000 nM) and set up time-dependent cell cycle analysis by PI-stain flow cytometry. Accumulation of cells in the G1/G0, S or G2/M phases was monitored 6, 24 and 72 hours after application of either agent.

Of interest, NVP-BGT226 produced a shift of cells from G2/M and S-phase to the G1/G0 phase – but also markedly increased the proportion of a sub-G0/G1 fraction, indicating dead/apoptotic cells, with a proportion of 50% (MOLM14, Figure 3C) and 41% (K562, Figure 3D) 72 hours after treatment. In contrast, NVP-BEZ235 lead to profound und sustained accumulation of cells in the G0/G1 phase – with only 19% (MOLM14) and respectively 13% (K562) of cells rendering into the sub-G0/G1 fraction after 72 hours of incubation.

Even more, when using high doses (i.e. 10 000 nM), which kill virtually all cells exposed to NVP-BGT226, strong accumulation of MOLM14 as well as K562 cells within the G1/G0 fraction was observed for NVP-BEZ235-treated cells (sub-G1/G0 fractions of only 35% (MOLM14) and 17% (K562)). This observation argues for a potent and sustained cell cycle arrest caused by NVP-BEZ235 in these cell lines.

**Figure 4:**
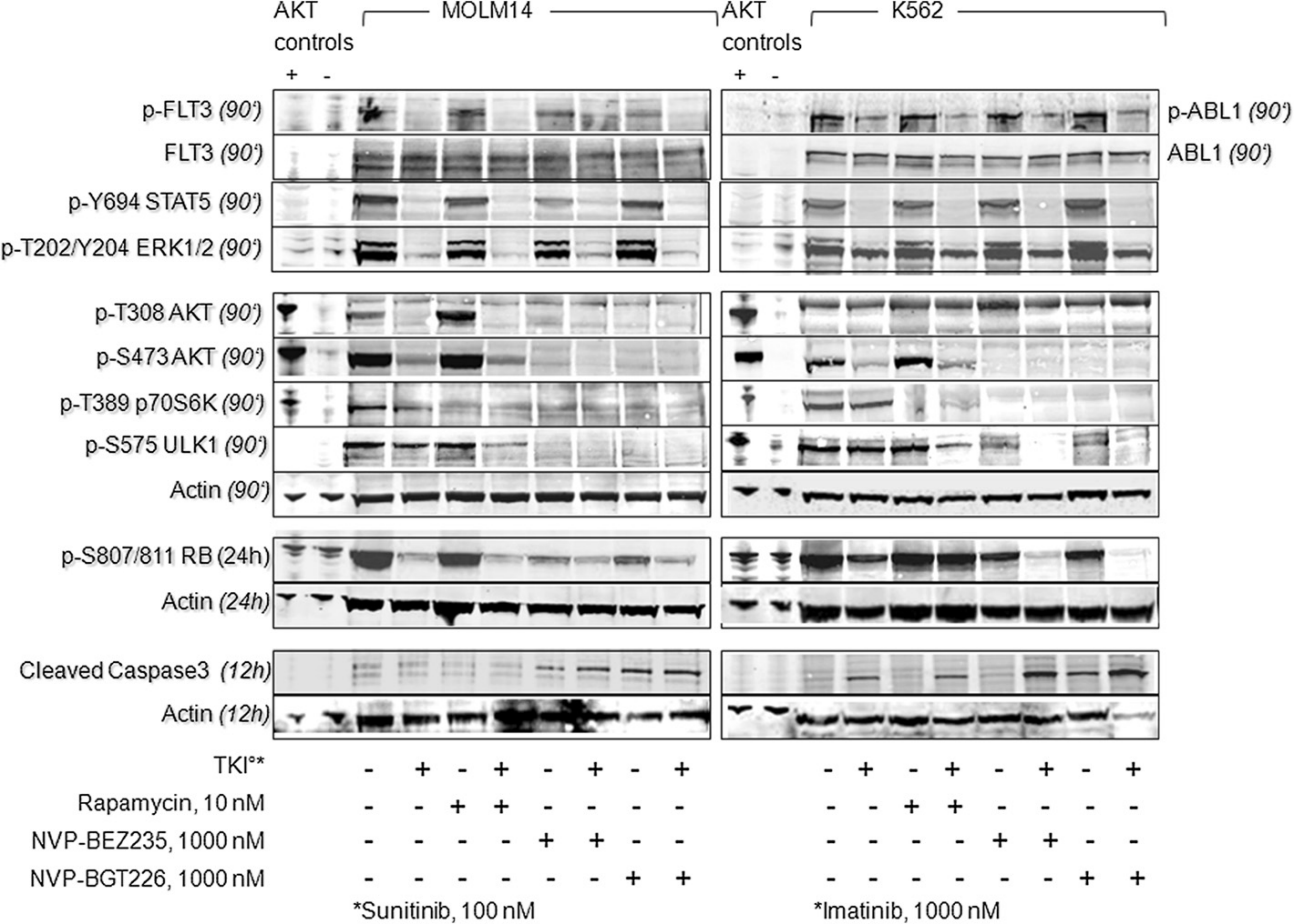
**Mutant-TK mediated AKT signaling pathways are potently and globally suppressed by dual PI3K/MTOR inhibition.** Immunoblotting of equally loaded whole cell extracts reveals strong consecutive suppression of AKT signaling pathways in the acute leukemia cell lines MOLM14 or K562, which harbor known autoactivating tyrosine kinase (TK) mutations (MOLM14: *FLT3* ITD; K562: *BCR/ABL1*). TKI and specific MTORC1 inhibition by rapamycin reveal a differential inhibition profiles. Jurkat cells are used as positive controls for activated AKT signaling; PI3K-inhibited Jurkat cells (treated with LY294002) serve as negative controls. Actin blotting is used as a loading control.

For validation purposes, we set up immunoblotting experiments using whole cell lysates extracted from MOLM14 or K562 cells treated with either NVP-BGT226 or NVP-BEZ235 (Figure 4). For comparative analysis, additional lysates from cells treated with an *ABL1* or *FLT3* tyrosine kinase inhibitor (imatinib for K562 *BCR-ABL1* cells, sunitinib for MOLM14 *FLT3* ITD cells) as well as rapamycin were used.

NVP-BGT226 as well as NVP-BEZ235 potently suppressed phosphorylation of AKT at Ser473 as well as Thr308. As expected, these compounds did not affect phosphorylation of *FLT3* or *ABL1* tyrosine kinases, nor did they affect phosphorylation patterns of MAPkinases (ERK1/2) or STAT5, which are known downstream signaling targets activated by oncogeneic TK mutations such as *FLT3* ITD or *BCR-ABL1*.

It has to be noted, that basal phosphorylation levels of T308-AKT in MOLM14 and K562 cells were relatively weak to absent – which will be discussed later in more detail using an isogenic Ba/F3 mutant-TK model.

We furthermore probed for downstream signaling targets of AKT: Activation of autophagy cascades (via ULK1) and decreased cell cycle progression in G1 (via dephosphorylation of p70S6K and RB) was similarly seen for both agents – and correlated best with dephosphorylation of AKT at Ser473. In contrast, only NVP-BGT226 treated cells managed to override halt of cell growth and induction of autophagy to induce apoptosis in a cell cycle independent manner as indicated by increased cleavage activity at caspase 3 in both tested cell lines.

The western blot experiments hereby support the findings taken from the cell-based assays for cellular proliferation and induction of apoptosis for both agents.

On a side note, comparative analysis of a specific MTORC1 inhibitor (rapamycin) revealed consecutive dephosphorylation of p70S6K – but no concomitant meaningful inhibition of ULK1 or RB phosphorylation, no cleavage of caspase 3 and no effect on *FLT3* or *ABL1* signaling in the tested dose. Importantly, rapamycin did not suppress AKT phosphorylation – but activates AKT via a negative feed back loop mechanisms as previously reported [24,26]. This may counteract clinical efficacy of single MTORC1 inhibition.

For TKI-treated cells we confirmed potent inhibition of the corresponding tyrosine kinase, as well as downstream signaling pathways including MAPKinases, STATs as well as AKT [35,36]. However, dephosphorylation of the AKT pathway was less pronounced compared to STAT5 or ERK1/2 inhibition, leaving downstream signals (partially) phosphorylated. This observation argues for a potential rescue mechanism of TKI monotherapy, which may be overridden by combination approaches: As indicated in our immunoblot panel, a combination of TKI with PI3K/AKT signaling inhibitors, such as rapamycin or dual PI3K/MTOR inhibitors, potently and globally suppresses AKT signaling pathways as well as mutant-TK mediated pathways including MAPKinases and STAT5 signaling.

**Figure 5:**
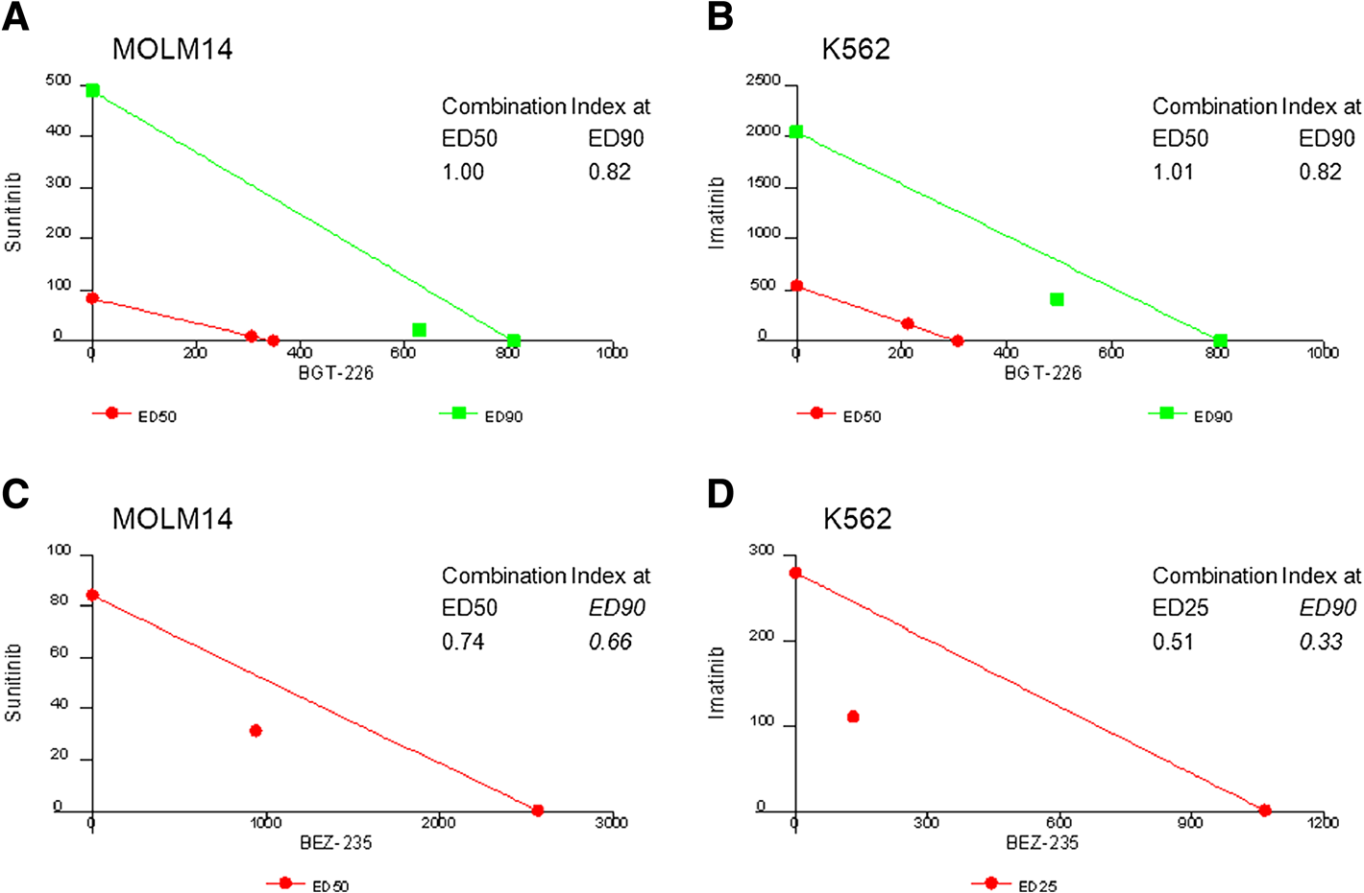
**Isobologram analyses of combined dual PI3K/MTOR plus TKI treatment in mutant-TK activated leukemia cells.** Co-treatment of specific TKI (sunitinib to target *FLT3* ITD in MOLM14 cells (**A** and **C**), imatinib to target *BCR-ABL1* in K562 cells (**B** and **D**) with either NVP-BGT226 (**A** and **B**) or NVP-BEZ235 (**C** and **D**) reveal additive to synergistic proapoptotic effects for the combination treatment: Isobolograms are provided, showing experimental points that fall on or below the predicted line of additive effect, indicating additive to superadditive effects for all tested endpoints. Calculations of combination indices (CI) provide a mathematical number to describe the degree of synergy for the respective endpoint.

To provide a mathematical tool to describe the combination effect of two agents, we performed fixed ratio dilution experiments to create isobolograms using a method of Chou and Talalay [37]. Cells were treated with the single agents and fixed ratios of NVP-BGT226 or NVP-BEZ235 plus sunitinib (MOLM14 cells) or imatinib (K562 cells) to assess for induction of apoptosis. This was used to create isobolograms (Figure 5). Combination of NVP-BGT226 with sunitinib in MOLM14 cells (Figure 5A) resulted in an experiment point that falls to the left of the predicted line of additive effect when taking ED90 (i.e. 90% apoptotic cells) as the experimental end point (indicating a superadditive effect). Similar results were achieved for NVP-BGT226 combined with imatinib in K562 cells with an experiment point lying on (ED50) or falling to the left (ED90) of the predicted line of additive effect (Figure 5B).

Calculation of combination indices (CI) revealed a CI close to 1 (i.e. additive effect) for ED50s in both cell lines and a CI < 1 for ED90 – indicating synergy (i.e. superadditive effects).

Due to the moderate proapoptotic effect of NVP-BEZ235 when administered as single agent, calculation of isobolograms and resultant CIs were restricted to ED25-50 concentrations for NVP-BEZ235+TKI combinations. Nevertheless, a strong synergistic effect was revealed for both combinations of NVP-BEZ235 plus sunitinib in *FLT3* ITD positive MOLM14 cells (Figure 5C), or NVP-BE235 plus imatinib in *BCR-ABL1* positive K562 cells (Figure 5D) with CIs well smaller than 1. Additionally, estimated ED90s are provided along with each figure as well. These findings indicate that a combination approach may override the G1/G0 arrest observed for NVP-BEZ235 monotherapy – which is supported by increased cleavage of caspase 3 in the western immunoblot experiments when combined with TKI (Figure 4).

### Leukemia-driving tyrosine kinase mutations trigger consecutive AKT serine phosphorylation of codon 473 and threonine phosphorylation of codon 308

In order to minimize cell type specific off-target effects to validate our findings for the mutant *FLT3* ITD cell line MOLM14 and the *BCR-ABL1* positive cell line K562, we established an isogenic Ba/F3 cell line model transfected with AKT-autoactivating *FLT3* ITD or *BCR-ABL1* mutations. We further comparatively extended our studies to additional leukemia-associated mutant-TK (a full list of tested mutant-TK Ba/F3 cell transfectants are provided with Table 1).

Immunoblotting for phospho-AKT was performed after successful transfection and weaning of IL3-dependent growth (true autoactivating TK mutations result in IL3-independent growth) and found that AKT activation increases after transfection of plasmid vectors encoding for a *FLT3* ITD, *FLT3* D835V, *KIT* D816Y or *BCR-ABL1* isoform.

**Figure 6:**
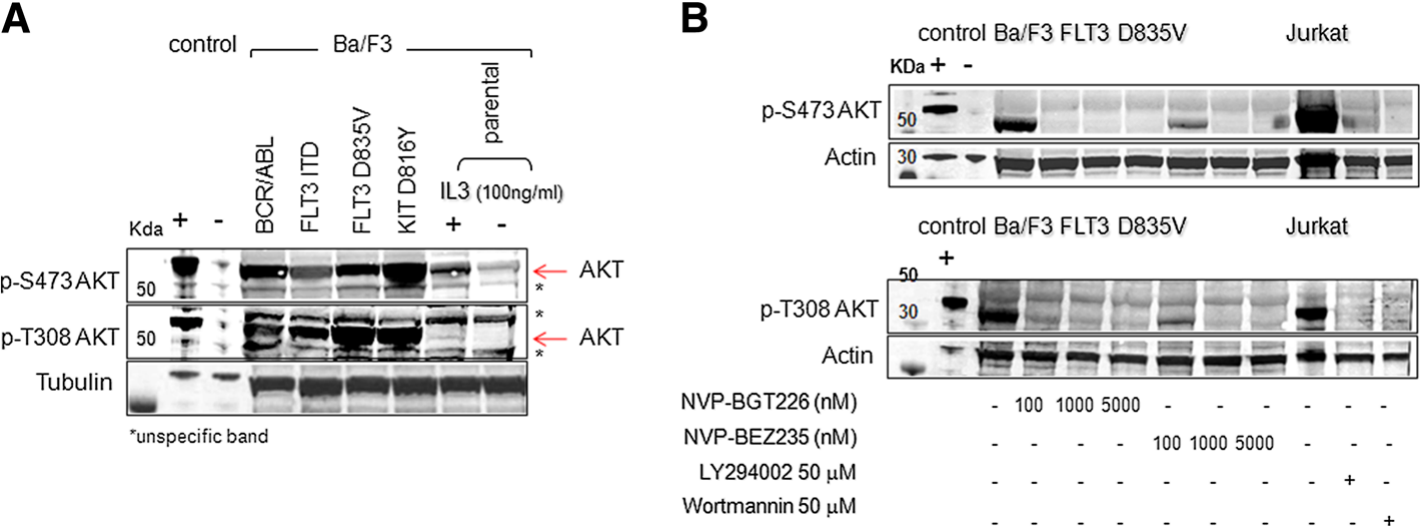
**Western immunoblot analyses of mutant-TK Ba/F3 cells as a cell model for activated AKT-signaling.** (**A**) Transfection of leukemia-driving mutant-*KIT, FLT3* or *BCR-ABL1* isoforms into a hematopoietic IL3-dependent Ba/F3 cell line leads to global increase of AKT phosphorylation as assayed by immunoblotting of whole cell extracts. Phosphorylation of Ser473 and Thr308 increases in response to IL3 stimulation - and further augments in cells transfected with a gain-of-function mutant-TK. Tubulin blotting is used as loading control. (**B**) An immunoblot experiment with Ba/F3 cells transfected with a *FLT3* D836V mutation treated with NVP-BGT226 or NVP-BEZ235 and probed for phosphorylation patterns of AKT is shown. Jurkat cells are used as positive controls. Jurkat cells treated with PI3K inhibitors (Wortmannin, LY294002) serve as negative controls for AKT phosphorylation. Actin blotting serves as a loading control.

While cytokine-starved parental BaF3 cells did only reveal moderate, if any, phosphorylation levels of AKT, IL3-stimulated or oncogene-transfected Ba/F3 cells did globally activate AKT on codons Thr308 as well as Ser473. Notably, TK-mediated activation of AKT was by far more pronounced compared to physiologic, cytokine (IL3)-mediated activation of AKT (Figure 6A).

We tested our model by treating Ba/F3 cells transfected with the gain-of-function *FLT3* D835V mutation with either NVP-BGT226 or NVP-BEZ235 and probed for T308- or S473-phosphorylated AKT isoforms in a western immunoblot using whole cell lysates. Both inhibitors potently and globally suppressed AKT phosphorylation of initially maximally activated AKT (Figure 6B). Jurkat cells treated with well established PI3K inhibitors (Wortmannin, LY294002) served as controls.

### NVP-BGT226 displays antiproliferative and proapoptotic activity in mutant-tyrosine kinase mediated AKT-activated Ba/F3 isogenic cells

**Figure 7:**
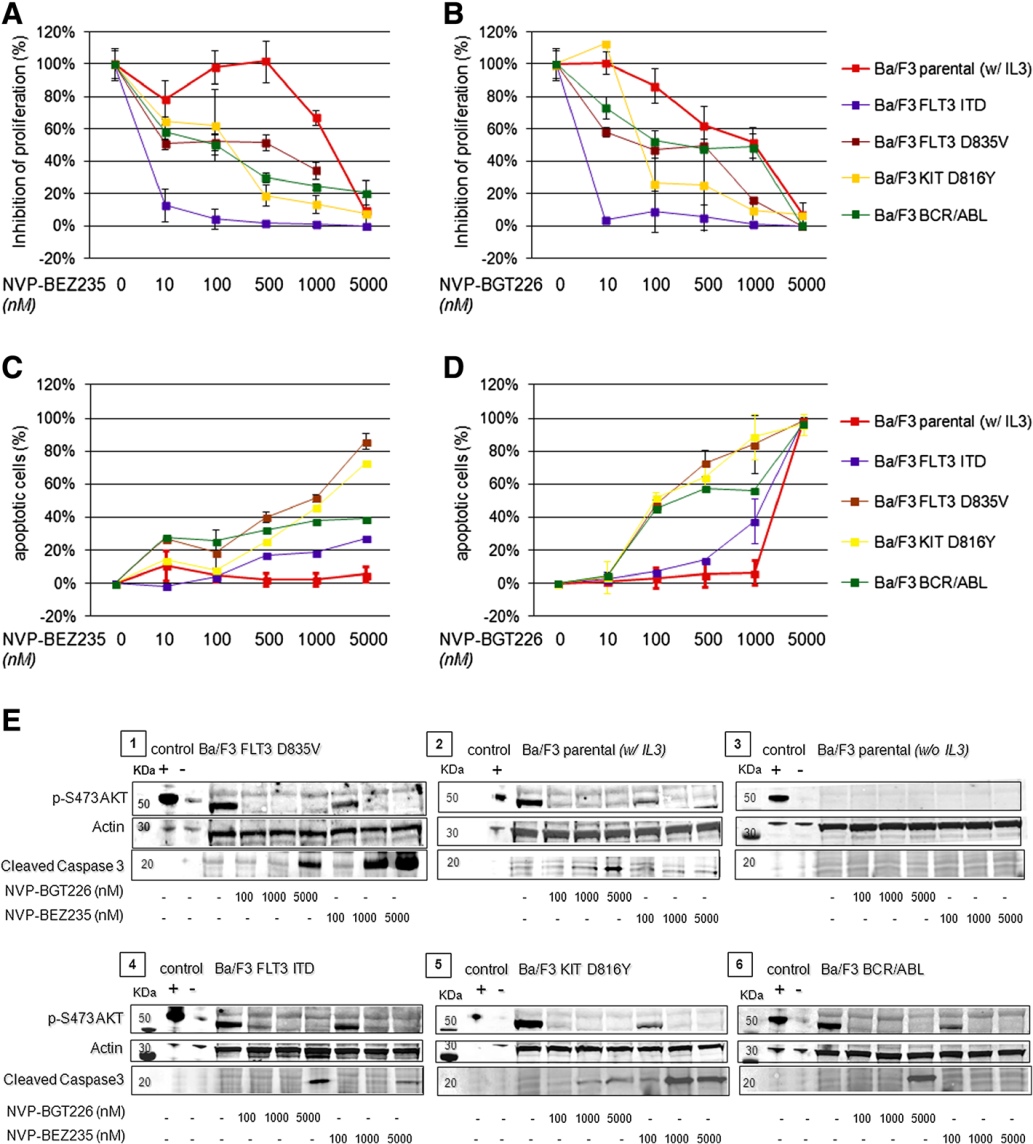
**Mutant-tyrosine kinases sensitize Ba/F3 cells to dual PI3K/MTOR inhibition.** Ba/F3 cells transfected with *KIT* D816Y*, FLT3* ITD, D835V or *BCR-ABL1* isoforms were exposed to NVP-BEZ235 (**A**) or NVP-BGT226 (**B**) in a dose-dependent manner. Dual PI3K/MTOR inhibition results in transfectant- and inhibitor-specific inhibition of cellular proliferation (XTT assay) with a higher sensitivity of cells transfected with leukemia-driving mutant-TK (*KIT, FLT3*, *ABL1*) isoforms compared to the (IL3-stimulated) parental cell line. When setting induction of apoptosis as the experimental endpoint, NVP-BEZ235 (**C**) was less effective compared to NVP-BGT226 (**D**), in all tested transfectants. IC50s, estimated by linear dose regression analysis, are provided along with several more transfectants in Table 1. (**E**) On the protein level, IL3-stimluated or mutant-TK Ba/F3 transfected cells show similar potent AKT dephosphorylation of Ser473 but cell strain specific cleavage of caspase 3 upon NVP-BGT226 or NVP-BEZ235 exposure. Parental, cytokine-depleted Ba/F3 cells do not express phosphorylated AKT and neither NVP-BGT226 nor NVP-BEZ235 induces cleavage of caspase 3.

We next utilized our Ba/F3 model to evaluate the mutant-TK specific antiproliferative effect of either NVP-BGT226 or NVP-BEZ235 in an isogenic cellular background. Both agents revealed compound-specific – but also distinct mutation-specific activity, with the parental cell line (stimulated with IL3) being the least sensitive for both tested agents (estimated IC50s by regression dose effect analysis ~2400 nM for NVP-BEZ235 and ~800 nM for NVP-BGT226). *BCR-ABL1*, *FLT3* D835V and *KIT* D816Y transfectants displayed an intermediate sensitivity pattern (estimated IC50s for NVP-BEZ235 ~10-130 nM and ~65-180 nM for NVP-BGT226) whereas *FLT3* ITD demonstrated high sensitivity for both agents with IC50s below 10 nM. Representative dose vs. effect graphs are shown in Figure 7A/B. A summary of achieved IC50s is provided in Table 1 - together with additional (mutant-) TK isoforms tested.

**Table 1:** **Isogenic Ba/F3 model: apoptosis and proliferation assays *- IC50s***

**NVP-BEZ235**		**IC50 (nM)**		**NVP-BGT226**		**IC50 (nM)**	
***Induction of apoptosis***		***At 1000 nM (% apopt.)***	***At 5000 nM (% apopt.)***	***Induction of apoptosis***		***At 1000 nM (% apopt.)***	***At 5000 nM (% apopt.)***
*Parental (w/ IL3)*	Not reached*	*3*	*6*	*Parental (w/ IL3)*	*1821*	*7*	*99*
*KIT* WT	1396	32	*85*	*KIT* WT	463	*48*	*97*
*KIT* D816V	Not reached	*4*	*23*	*KIT* D816V	1791	*7*	*99*
*KIT* D816F	1244	*40*	*87*	*KIT* D816F	258	*87*	*89*
*KIT* D816Y	1363	*5*	*73*	*KIT* D816Y	118	*89*	*96*
*FLT3* WT	8296	*38*	*40*	*FLT3* WT	345	*41*	*99*
*FLT3* S451F	741	*49*	*70*	*FLT3* S451F	125	*93*	*99*
*FLT3* ITD	Not reached	*19*	*27*	*FLT3* ITD	1079	*38*	*98*
*FLT3* K663Q	Not reached	*17*	*16*	*FLT3* K663Q	1111	*26*	*98*
*FLT3* D835V	458	*52*	*86*	*FLT3* D835V	163	*84*	*98*
*FLT3* D835Y	Not reached	*19*	*28*	*FLT3* D835Y	1073	*33*	*98*
*FLT3* N841H	Not reached	*19*	*22*	*FLT3* N841H	1206	*18*	*99*
*BCR*/*ABL1*	Not reached	*38*	*39*	*BCR*/*ABL1*	262	*56*	*97*
***Inhibition of proliferation***		***At 10 nM (% alive)***	***At 100 nM (% alive)***	***Inhibition of proliferation***		***At 10 nM (% alive)***	***At 100 nM (% alive)***
*Parental (w/ IL3)*	*2398*	*89*	*98*	*Parental (w/IL3)*	*828*	*100*	*86*
*KIT* WT	6	*12*	11	*KIT* WT	6	24	13
*KIT* D816V	8	*40*	38	*KIT* D816V	153	110	47
*KIT* D816Y	72	*64*	62	*KIT* D816Y	65	112	26
*FLT3* WT	7	*33*	28	*FLT3* WT	40	66	27
*FLT3* K663Q	91	*57*	51	*FLT3* K663Q	277	79	52
*FLT3* ITD	7	*13*	4	*FLT3* ITD	3	4	9
*FLT3* D835V	10	*50*	51	*FLT3* D835V	141	58	47
*FLT3* D835Y	5	*37*	30	*FLT3* D835Y	35	65	33
*FLT3* N841H	9	*43*	47	*FLT3* N841H	89	110	38
*BCR*/*ABL1*	131	*58*	50	*BCR/ABL1*	183	73	52

*IC50s not reached (> 5 000 nM).

When testing for induction of apoptosis, NVP-BGT226 proved to be highly potent in virtually all tested cell lines, with transfectant-specific IC50s raging from ~120-1800 nM (again, the parental IL3-stimulated cell line being the least sensitive). In contrast, the high capacity to inhibit cellular proliferation for NVP-BEZ235 did not similarly translate into potency to induce apoptosis for all tested transfectant cell lines. Importantly*,* Ba/F3 *FLT3* ITD cells, which were highly inhibited with regard to cellular proliferation, did only show moderate induction of apoptosis towards NVP-BEZ235 (IC50 not reached up to tested doses of 10 000 nM, proportion of apoptotic cells at 5000 nM: 27%). In analogy, *BCR-ABL1* transfected cells failed to achieve IC50 as well, with a proportion of 39% apoptotic cells at 5000 nM). These findings are in line with our results for the corresponding tested human leukemia cell lines. Notably, other transfectants (e.g. *FLT3* D835V and *KIT* D816Y) retained some level of sensitivity with regard to induction of apoptosis. Representative dose vs. effect graphs are shown in Figure 7C/D. A full list of IC50s for both agents and additionally tested mutant-TK Ba/F3 cells are provided with Table 1.

We confirmed our observations at the protein level and treated Ba/F3 parental (+/− IL3), *FLT3* ITD, *FLT3* D835V, *KIT* D816Y or *BCR-ABL1* transfected cells with NVP-BGT226 or NVP-BEZ235 to probe whole cell lysates for AKT phosphorylation in an immunoblot. Dual inhibition of PI3Kinases and MTOR1/2 lead to potent AKT dephosphorylation of initially activated AKT in IL3-stimulated or mutant-TK activated cells in the low nanomolar range (Figure 7E). This went along with the observed antiproliferative effects for both agents on the cellular level. In line with our cellular apoptosis assays, immunoblotting for cleaved caspase 3 as an indicator for induced apoptosis again revealed that higher doses are needed to induce programmed cell death in these cell lines (if any, when looking at NVP-BEZ235). These findings argue for a complex regulation of programmed cell death, which will need to be studied in more detail in future studies. One hypothesis may state that induction of apoptosis is mediated via Thr308: We observed a particular high phosphorylation pattern of Thr308 in cells transfected with the tyrosine kinase domain (TKD)-mutated *FLT3* D835V and *KIT* D816Y isoforms in our assays (see Figure 6). Interestingly these were the cell lines to display the highest rates of apoptosis after treatment. In contrast *BCR-ABL1* or *FLT3* ITD transfectants, presenting with comparably lower p-T308 AKT levels, were by far less sensitive towards NVP-BEZ235 with regard to induction of apoptosis (compare Figure 7C). These observations are in line with Thr308 phosphorylation levels seen in MOLM14 and K562 cell lines, which were relatively weak to absent (see also Figure 4).

### NVP-BGT226 displays antileukemic activity in native leukemia blasts treated *ex vivo*

To evaluate, whether our *in vitro* data derived from leukemia cell lines and mutant-TK cell line models translate into a clinically meaningful antiproliferative effect in native leukemia cells, we treated an acute leukemia sample taken from a patient suffering from *FLT3* mutant-TKD2 positive AML and a sample from a patient with AML tested negative for *FLT3* or *KIT* mutations with varying concentrations of NVP-BGT226 or NVP-BEZ235 and tested for the capacity to inhibit cellular proliferation *ex vivo* using an XTT-based assay. The *FLT3* TKD2 positive leukemia sample (pat. 556) revealed high sensitivity towards NVP-BGT226 as well as NVP-BEZ235 with calculated IC50s in the low nanomolar range in a dose-effect plot. In contrast the AML sample lacking mutant-TK isoforms (pat. 303) was virtual insensitive towards both agents with IC50s well above 5000 nM. Importantly, mononuclear cells extracted from an aspirate of a bone marrow donor (bm-donor 552) revealed a sensitivity profile of IC50s > 1000 nM for both compounds. Dose-effect plots were created for tested patient samples to calculate IC50s, which are provided in Table 2 along with AKT expression patterns (exemplary dose-effect plots to calculate IC50s is provided in Additional file 2: Figure S1A; patient characteristics are provided in Additional file 1: Table S1).

**Table 2:** **Leukemia models: *Comparison of response rates and AKT expression levels***

**Pt. Nr.**	**p-AKT (Thr308) expression**	**p-AKT (Ser473) expression**	**pan-AKT expression**	**Mean overall expression**	**Apoptosis BEZ235**	**Proliferation BEZ235**	**Apoptosis BGT226**	**Proliferation BFT226**
	***Normalised to mean expression of all donors***			***GeoMean (pT308+pS473+panAK)***	***IC50 (nM)***	***IC50 (nM)***	***IC50 (nM)***	***IC50 (nM)***
*538 (donor)*	0,8	0,7	0,8	**0,77**	Not reached	1779		
*554 (donor*)	0,9	1,0	0,7	**0,87**	Not reached	3814		
290	1,7	1,5	2,4	**1,82**	71	4		
368	1,9	2,7	1,5	**1,96**	3182	149		
527	0,8	1,9	1,3	**1,25**	6824	12		
528	2,4	2,5	1,5	**2,07**	371	12		
532	2,8	1,2	1,2	**1,59**	653	25		
*552 (donor)*	1,3	1,3	1,5	**1,34**		1019		1081
303	0,9	1,6	2,0	**1,38**		6142		5590
556	1,5	1,9	1,2	**1,48**		24		32

The findings of equipotent sensitivity profiles of NVP-BGT226 and NVP-BEZ235 with regard to inhibition of cellular proliferation in native AKT-activated leukemia cells are in line with our *in vitro* data provided above. Notably, the PI3K/AKT/MTOR pathway is a target of NVP-BGT226 as well as NVP-BEZ235 in native acute leukemia cells as verified in an immunoblot experiment for two patient samples with newly diagnosed acute leukemia (Additional file 2: Figure S1B, provided with the online version of the article). This further underlines and validates the herein described *in vitro* and *ex vivo* data rather than arguing for off-target effects.

Correlation of *ex vivo* responses to NVP-BGT226 and NVP-BEZ235 with AKT expression levels suggests that augmented activation of AKT (compared to healthy bone marrow donors), i.e. phosphorylation of Thr308 as well as Ser473 but not mere AKT protein levels, may be a requisite for inhibition of cellular proliferation in response towards dual PI3K/MTOR inhibition. Clearly, analysis of pan-AKT protein levels may not predict for response, as AKT expression was highest in the AML sample refractory towards both inhibitors (Table 2).

Next, we studied, whether NVP-BGT226 and NVP-BEZ235 are capable of inducing apoptosis in native leukemia samples. Leukemia blasts extracted from acute myeloid, promyelocytic or lymphoid leukemia with or without detectable TK mutations were treated with NVP-BGT226 or NVP-BEZ235 in dose dilution series and apoptosis was assessed by an Annexin V/PI stain.

In analogy to our *in vitro* data described before, both agents demonstrated variable apoptosis induction. Notably, NVP-BGT226 proved to be the more potent drug with high effectivity and IC50s in the lower nanomolar range in some patient samples (Table 2). Of note, native mononuclear cells derived from bone marrow donors revealed much higher IC50s for both agents. Analysis of AKT expression levels suggest that global activation of AKT with augmented phosphorylation of Ser473 as well as Thr308 beyond a baseline set as 1 on a normalised AKT expression scale is a prerequisite to predict response towards the dual PI3K/MTOR inhibition. However, this observation will need prospective verification on a larger patient cohort.

## Discussion

PI3K/AKT signaling controls key signaling pathways involved in the maintenance of cellular viability and proliferation in many cells and tissues. Not surprisingly, activation of AKT is increased in many human malignancies and gain-of-function mutations are frequently found within PI3K/AKT axis, especially in solid tumors, making the PI3K/AKT signaling pathway an attractive target for molecular therapeutics.

In acute leukemia, activating mutations in the PI3K/AKT signaling cascade are rare – but nevertheless, we and others have reported frequent activation of AKT (i.e. phosphorylation of Thr308 and Ser473): In this study, we demonstrate global phosphorylation of AKT in native acute leukemia samples. Average expression levels are thereby statistically significantly elevated compared to physiologic hematopoietic mononuclear cells derived from healthy donors. Moreover, augmented expression levels are exclusively found in the leukemia cohort.

The mechanisms of AKT activation in acute leukemia are only partially understood. One mechanism of constitutive phosphorylation of AKT can be explained by the presence of gain-of-function mutant tyrosine kinases, which are found in approximately 30-40% of adult AML and ALL. However, we did not find an exclusive correlation of phospho-AKT expression levels and the presence of TK mutations, suggesting other mechanisms, which render AKT autoactivated in leukemia cells. Evaluation of the triggering mechanisms are topic of ongoing research.

Globally targeting the AKT signaling pathways may be a promising approach to treat acute leukemia. We herein evaluated the antileukemic efficacy of the novel dual PI3K/MTOR inhibitor NVP-BGT226, a pan-PI3Kinase inhibitor also targeting the rapamycin-sensitive MTOR complex 1 as well as the rapamycin-insensitive MTOR complex 2.

Using defined cell line models, and primary leukemia patient as well as donor samples we studied the distinct effects of NVP-BGT226 on cellular proliferation, cell cycle progression and induction of apoptosis. Thereby we compared NVP-BGT226 to a second dual inhibitor, NVP-BEZ235, which is currently under investigation in a phase I study for relapsed/refractory ALL or AML (European Clinical Trials Database number: EUDRACT2011-005050-61).

Our cell models included cell lines with defined genomic alterations rendering the AKT signaling pathway autoactivated, i.e. (i) a PTEN-deficient acute T-lymphoblastic leukemia cell line (Jurkat), (ii) patient-derived leukemia cell lines with well described TK-mutations (MOLM14 harboring a *FLT3* ITD mutation and K562 harboring a *BCR-ABL1* fusion transcript mutation), (iii) engineered Ba/F3 cell lines transfected with mutant tyrosine kinases expressed in an otherwise isogenic cellular background and (iiii) native *ex vivo* acute leukemia cells, with or without a defined TK-mutation, derived from consented patients with newly diagnosed acute leukemia. In addition, we comparatively studied native physiologic mononuclear cells derived from bone marrow donors.

In PTEN-deficient Jurkat cells, NVP-BGT226 proved to potently inhibit cellular proliferation in the low nanomolar range. The sensitivity profile is thereby in the same range compared to the additionally tested dual PI3K/MTOR inhibitor, NVP-BEZ235.

It was previously noted, that the predominant antitumor effect of inhibitors of PI3K/AKT/MTOR signaling cascades is mediated via inhibition of cellular proliferation rather than induction of apoptosis [32,38,39]. Surprisingly however, NVP-BGT226 proved to have genuine proapoptotic efficacy – whilst the proapoptotic effect achieved by NVP-BEZ235 was, as expected by previous reports, at most moderate.

To model the effects of NVP-BGT226 and NVP-BEZ235 on mutant-TK triggered AKT activation, we chose two well established acute leukemia cell lines harboring a *FLT3* ITD mutation (MOLM14) or a *BCR-ABL1* mutation (K562). Similar to the findings for Jurkat cells, both inhibitors, proved to be highly potent in inhibiting cellular proliferation. However again, NVP-BEZ235 only moderately induced a meaningful proapoptotic effect, whereas NVP-BGT226 was a strong inducer of the programmed cell death machinery.

As the AKT pathway controls cell cycle checkpoints, we speculated that the discrepancy may be due to differential activity on the cell cycle compartment. And indeed, a strong and sustained G0/G1 arrest was observed for NVP-BEZ235 preventing cells to undergo apoptosis.

On the protein level, where both agents were similarly targeting downstream proteins controlling cell cycle progression (such as S6Kinases and RB) or ULK1-induced autophagy, only NVP-BGT226 was capable to override cell protective mechanisms to potently induce apoptosis.

We speculated that the cell cycle arrest induced by NVP-BEZ235 might be overcome by combination approaches: TKI, for which we demonstrated insufficient global suppression of AKT signaling pathways – but additional effects on alternative survival pathways such as MAPK and STAT signaling, may be an attractive molecularly defined partner to combine with dual PI3K/MTOR inhibitors. Indeed on the protein level, combination of TKI with either of the tested dual PI3K/AKT inhibitors efficiently and globally shut down AKT signaling pathways - as well as additional targets (ERK1/2, STAT5) triggered by mutant-TKs.

In an attempt to mathematically define the extend of combination efficacy, we established isobologram assays to compute combination indices (CI). Together, calculated CIs for TKI plus dual PI3K/MTOR inhibitor treatment were close to or smaller than 1, indicating an additive to superadditive (synergistic) effect for all tested endpoints.

Notably, combination of TKI with NVP-BEZ235 was capable to override cell cycle arrest seen for NVP-BEZ235 monotherapy to potently induce apoptosis in leukemia cells.

One might speculate that cell-type specific off-target effects may have prevented cells to undergo apoptosis. To confirm our findings, we established an isogenic Ba/F3 cell line model transfected with *FLT3* ITD (corresponding to MOLM14 cells) or *BCR-ABL1* (corresponding to K562 cells) mutations. NVP-BGT226 revealed high potency to inhibit cellular proliferation in the same range as NVP-BEZ235.

As expected, while meaningful proapoptotic effects were achieved by NVP-BGT226 in all cell strains, *FLT3* ITD and *BCR-ABL1* transfected Ba/F3 cells were only moderately sensitive towards NVP-BEZ235.

We additionally created several more Ba/F3 cell lines transfected with tyrosine kinases harboring known leukemia-driving gain-of-function mutations and tested for NVP-BGT226 and NVP-BEZ235 sensitivities. While NVP-BGT226 again displayed a beneficial pro-apoptotic profile for all tested transfectants, NVP-BEZ235 surprisingly retained meaningful proapoptotic activitiy in some cell strains. Two sensitive transfectants (harboring a *FLT3* D835V or *KIT* D816Y mutation) were immunoblotted – and showed higher elevated threonine 308 phosphorylation levels compared to *FLT3* ITD or *BCR-ABL1* transfected cells.

This observation may have far-reaching consequences: It is tempting to speculate that activation of the PI3K/AKT pathway is at least in part dependent on the specific type of TK gain-of-function mutation and that different gain-of-function mutations may display a very distinct pattern of activated PI3K/AKT signaling cascades. This again might influence the susceptibility of cells towards PI3K/AKT-targeted inhibitors. In this context, it is well described for TKI therapy of CML and GIST and has recently been shown for TKI therapy in acute leukemia as well, that resistance towards TK-inhibitors is often caused by secondary mutations within the tyrosine kinase domain (such as point mutations at *FLT3 D835*) of the respective tyrosine kinase [40]. Such mutations may activate AKT signaling, as previously demonstrated for imatinib-resistant GIST tumors [41], and sensitize cells towards targeted therapies.

We tested this theory using two cell models comparing primary TK-sensitive mutations with secondary TK-insensitive mutations: The first model consists of a mast cell leukemia cell line (HMC1.1), which harbors an imatinib-sensitive *KIT* V560G mutation – and a derivative sister cell line (HMC1.2), which is characterized by a secondary activation loop *KIT* D816V mutation, rendering the cells insensitive towards imatinib [42,43]. Additionally we tested the GIST solid tumor cell line GIST882 (harboring an imatinib-sensitive *KIT* K642E mutation) [44] with a second cell line, which was established from a patient with relapsing GIST under imatinib therapy (GIST48) [45]. This cell line harbors a primary homozygous juxtamembrane *KIT* mutation (V560D) plus a secondary heterozygous imatinib-insensitive activation loop mutation (D820A).

Indeed, in our experiments, NVP-BEZ235 as well as NVP-BGT226 potently induced apoptosis irrespective of the sensitivity profile towards TKI – with NVP-BGT226 again being the more potent inhibitor (data provided as Additional file 3: Figure S2 with the online version of the article). Together, dual PI3K/MTOR inhibitors such as NVP-BGT226 or NVP-BEZ235 may be of special clinical value in the desperate case of tumor progress due to TKI-resistance, which is an ever increasing problem in the treatment of relapsed acute leukemia. The underlying molecular mechanisms determining the susceptibility of cells towards induction of apoptosis as well as sensitivity towards NVP-BGT226 or NVP-BEZ235 (e.g. higher binding affinities and alternative (unknown) targets) is elusive and will need to be answered in future studies.

Most importantly however, we did show that dual inhibition of pan class I PI3Kinases plus MTOR1/2 complexes does translate into a genuine antiproliferative but also proapoptotic effect in native leukemia cells treated *ex vivo* – with NVP-BGT226 being the more potent drug with regard to induction of apoptosis. Augmented phosphorylation of AKT rather than mere expression of AKT protein levels seemed to be a prerequisite for treatment response. However, this observation will need prospective validation. Furthermore, efficacy was not restricted to leukemia samples with identified genomic mechanisms of AKT activation (such as tyrosine kinase mutations), suggesting alternative mechanisms of activation yet to be identified.

Of note, among the native leukemia samples treated successfully *ex vivo* with either agent were cases from patients with poor prognostic features lacking effective therapeutic options. For example, both agents were effective in AML with mutant *FLT3*, including a patient with TKI-resistant *FLT3* ITD (B1 sheet)-positive AML [46] who had relapsed after allogeneic stem cell transplantation.

Other refractory AML cases with *ex vivo* sensitivity of cells to PI3K/MTOR inhibition included a relapsed elderly patient with *MLL*-rearranged AML. In this context, it has been shown that *MLL* rearrangements associate with high *EVI1* expression, which predicts for dismal prognosis [47]. Further, Yoshimi and colleagues recently have demonstrated that *EVI1* activates AKT signaling due to loss of PTEN activity [48]. As there are currently no effective therapy options for treatment of *EVI1*-associated AML, targeting the PI3K/AKT/MTOR pathway may be particularly of interest.

Preliminary data of an early phase I trial of NVP-BEZ235 in the treatment of advanced unresectable solid tumors demonstrated good tolerability with no dose-limiting toxicities. Notably, hematologic side effects were seen – but were mild to moderate with reversible anemia after treatment discontinuation [49]. Currently, a study evaluating efficacy of NVP-BEZ235 in acute leukemia is recruiting (European Clinical Trials Database number EUDRACT2011-005050-61).

In our studies, NVP-BGT226 proved to be the more effective agent with regard to antileukemic efficacy. *Ex vivo* treatment revealed IC50s in the nanomolar or lower micromolar range and thus NVP-BGT226 may be an attractive agent for targeted treatment of acute leukemias.

A very recent phase I study evaluating NVP-BGT226 in advanced solid tumors demonstrated variable antitumor activity [50]. In this context, another recent report demonstrated that NVP-BGT226 results in cell cycle arrest in pancreatic cancer cell lines [51], which is in clear contrast to our findings. This may argue for the rather low antitumor efficacy reported in the above mentioned phase I trial in advanced solid tumors. Our data clearly states a differential biological behavior of acute leukemia cells with regard to regulation of cell growth, cell cycle progression and induction of apoptosis, which may still support specific clinical testing of NVP-BGT226 in acute leukemia.

Moreover, in our studies, normal mononuclear cells were significantly less inhibited by dual PI3K/MTOR inhibition than leukemia cells, indicating a therapeutic gap of these agents in the treatment of acute leukemia without significant suppression of normal hematopoiesis. Nevertheless, as NVP-BGT226 targets physiologic cells in the highest tested doses, clinical evaluation will need to address potential side effects on the hematopoietic progenitor/stem cell pool. However, even in the case of significant stem cell suppression, NVP-BGT226 may still serve as an attractive agent for bridging-to-transplant strategies or allogeneic transplant conditioning regimens – especially for high-risk or elderly patients lacking other options.

## Conclusion

In summary, dual PI3K/MTOR inhibition is highly effective against acute leukemia cells, both *in vitro* as well as *ex vivo*. This efficacy extends to leukemia blasts from patients with high-risk features. Notably, the novel dual PI3K/MTOR inhibitor NVP-BGT226 reveals extraordinary potency to inhibit proliferation as well as to induce apoptosis in the nanomolar range against a broad range of cell lines and *ex vivo* leukemia samples tested. Furthermore, NVP-BGT226 did not induce G1/G0 cell cycle arrest seen for other PI3K inhibitors, such as NVP-BEZ235 in our studies, making NVP-BGT226 a highly promising agent for clinical testing in acute leukemia. This may include combination approaches as well as targeted therapy of TKI-resistant leukemias. Based on our studies, clinical evaluation of this agent for targeted treatment of acute leukemia subtypes is strongly indicated.

## Methods

### Cell Culture

Ba/F3 cell lines were obtained through the American Type Culture Collection (ATCC, Manassas, VA). The MOLM14 cell line was acquired through the Fujisaki Cell Center (Okayama, Japan). The MLL-AF9 fusion positive acute monocytic leukemia cell line MOLM-14 harbors a heterozygous *FLT3* ITD mutation [52].

The T-lymphoblastic cell line Jurkat and the CML blast crisis cell line K562 were obtained from the Deutsche Sammlung für Mikroorganismen und Zellkulturen (DSMZ, Braunschweig, Germany).

The human mast cell leukemia cell line HMC1.1, harboring an imatinib-sensitive KIT V560G mutation [42], and the sister cell line HMC1.2, harboring an additional imatinib-insensitive KIT D816V mutation [43] were provided by Prof. Heinrich, OHSU, Oregon. The GIST tumor cell lines GIST48 and GIST882 were kindly provided by Dr. Kopp, (University of Tübingen, Germany). GIST882 is harboring a *KIT* K642E mutation [44]; GIST48 was established from a patient with relapsing GIST under imatinib therapy (GIST48). This cell line harbors a primary juxtamembrane *KIT* mutation plus a secondary imatinib-insensitive mutation in the kinase domain [45].

Cells were cultured in RPMI 1640, supplemented with 10% fetal bovine serum (GIBCO/Invitrogen, Darmstadt, Germany or Biochrom AG, Berlin, Germany), 1% penicillin G (10,000 units/mL), and streptomycin (10,000 μg/mg) and 2 mmol/L L-glutamine. In addition, parental Ba/F3 cells were supplemented with 10 ng/ml of mouse-IL3. Negativity for mycoplasma contamination was confirmed using the pluripotent PCR Mycoplasma test Kit (AppliChem, Darmstadt, Germany). Cell lines harboring a mutant *KIT, FLT3* or *BCR-ABL1* were sequence confirmed.

### Patient specimens

Bone marrow aspirate and peripheral blood samples from consented patients with acute leukemia as well as samples from healthy blood and bone marrow donors were collected in 5000 U heparin with the approval of the ethics committee of the Medical Faculties of the University of Tübingen or the University of Ulm. Mononuclear cells were isolated by Ficoll-Hypaque density gradient fractionation. Cells were cultured in DMEM medium, supplemented with 20% fetal bovine serum (GIBCO/Invitrogen, Darmstadt, Germany or Biochrom AG, Berlin, Germany), 1% penicillin G (10,000 units/mL), and streptomycin (10,000 μg/mg) and 2 mmol/L L-glutamine.

### Antibodies and reagents

The dual pan class I PI3K AND MTOR complex 1 and 2 inhibitors NVP-BEZ235 [28] and NVP-BGT226 [27], two imidazo[4,5-c]quinoline derivatives competitively binding to the ATP-binding cleft of these enzymes were provided by Novartis (Basel, Switzerland). Stock solutions were created according to the manufacturer’s instructions. Rapamycin and the PI3K inhibitors LY294002 and Wortmannin were obtained from Cell Signaling (Danvers, MA). The TKI dasatinib (formerly BMS-354825) and sunitinib (formerly SU11248) were obtained from the University of Tübingen Hospital Pharmacy and dissolved in DMSO to create 10 mmol/L stock solutions and stored at −20°C.

Rabbit anti-panAKT, panFLT3, panABL1 or anti-cleaved caspase 3 antibodies were used at a 1:500 to 1:1000 dilution. Rabbit anti-phospho-AKT antibodies detecting phosphorylated isoforms of T308-AKT, S473-AKT, S807/811-RB, S575-ULK1, T389-p70S6K, Y694-STAT5, FLT3, ABL1 or T202/Y204-ERK1/2 were used at a 1:250 to 1:1000 dilution. An anti-actin mouse monoclonal antibody was used as a loading control. All antibodies, if not otherwise indicated, were purchased from Cell Signaling Technology.

As controls for AKT Thr308- and Ser473-phosphorylation we used Jurkat cells untreated (phosphorylated positive control) or treated with LY294002 or Wortmannin (negative controls lacking T308/S473 phosphorylation).

Infrared dye-conjugated secondary goat anti-rabbit and anti-mouse antibodies to use in a LI-COR® imaging detection system were used according to standard protocols (LI-COR Biosciences, Lincoln, NE). For flow cytometry studies, fluorescent dye-conjugated secondary goat anti-rabbit or anti-mouse antibodies were used according to standard protocols. Cell Signaling anti-rabbit IgG(H+L),F(ab’)2Fragment Alexa Fluor conjugate antibodies were used to assess expression levels provided in Table 2. The Invitrogen Zenon Alexa Fluor labeling kit was used for expression levels provided in Additional file 1: Table S1.

### Immunoblotting

Cell pellets were lysed with 100 to 150 μl of protein lysis buffer (50 mmol/L Tris, 150 mmol/L NaCl, 1% NP40, 0.25% deoxycholate with added inhibitors aprotinin, AEBSF, leupeptin, pepstatin, sodium orthovanadate, and sodium pyruvate, respectively phosphatase inhibitor cocktails „2“ and „1“ or „3“ (Sigma, St. Louis, MO). Protein from cell lysates (75 to 200 μg protein) was used for whole cell protein analysis after denaturing by Western immunoblot assays using a BioRad Criterion system. Nonspecific binding was blocked by incubating the blots in nonfat dry milk or BSA. Primary antibodies were incubated for one hour or over night, followed by several washes of Tris-buffered saline (TBS) containing 0.005% Tween 20. The appropriate secondary antibody was applied for 30‘, followed by several washes. Antibody-reactive proteins were detected using a LI-COR Odyssey® fluorescence optical system.

### Immunophenotyping

Intracellular (phospho-)AKT protein expression levels were assayed as follows: Cells were fixed and permeabilized using the Fix & Perm® Fixation and Permeabilization kit (ADG-An der Grub Bioresearch, Kaumberg, Austria). Unlabeled primary AKT antibodies were added in a 1:1000 dilution to the cell suspension and incubated for 1 hour at room temperature followed by PBS washing and resuspension. Fluorescent dye-conjugated secondary antibodies were added in a 1:10 000 dilution and cells were incubated for 30 min at room temperature. After rinsing and resuspension, (phospho-)AKT protein expression levels were assayed using a FACScalibur® flow cytometer loaded with CellQuest® analysis software (BD, Heidelberg, Germany).

### Site-directed mutagenesis and generation of a Ba/F3 cell line expressing KIT, ABL1 or FLT3 isoforms

To compare constitutive activation of AKT mediated by autoactive tyrosine kinase signaling in a homologous cellular background, an isogenic cell model (Ba/F3) expressing different human tyrosine kinase mutations was established. An IL3-dependent murine pro-B cell line (Ba/F3) was transfected with plasmid vectors containing cDNA of human (mutant) *FLT3* and *KIT* isoforms, as well as the *BCR/ABL1* fusion mutation isoform. Gain-of function tyrosine kinase mutations lead to factor-independency.

Site-directed mutagenesis and generation of a Ba/F3 cell lines stably expressing mutant *KIT* D816V, D816Y, *FLT3* ITD, D835V, D835Y, K663Q, *BCR/ABL1* and *FLT3* wildtype was previously performed as described before [36,53-55].

*FLT3* S451F cDNA cloned into a pCMVneo plasmid vector [53] was generously provided by Dr. Fröhling, University of Ulm, Germany. *KIT* wildtype cDNA cloned into a pJP1563 plasmid vector was obtained from the DNASU Plasmid Repository at the Biodesign Institute of the Arizona State University (ASU). Lipofection transfection into the parental Ba/F3 cell line was performed to stably express *KIT* wildtype or mutant *FLT3* S451F by double selection for neomycin (pCMVneo plasmid), blasticidin (pJP1563 plasmid) or gentamicin (G418; all other plasmids) resistance and IL-3-independent growth. The Ba/F3 *KIT* wildtype cell line was cultured using recombinant human stem cell factor (SCF/KIT Ligand, R&D, Minneapolis, MN) as a growth supplement.

### Apoptosis and proliferation assays

Cells were treated in dilution series with the respective small molecule inhibitor.

Translocation of phosphatidylserine from the inner to the outer leaflet of the plasma membrane as an early indicator of apoptosis was analyzed using an Annexin V-based assay (Immunotech, Marseilles, France) and a FACScalibur® flow cytometer loaded with CellQuest® analysis software (BD, Heidelberg, Germany) [35].

Cellular proliferation was measured using an 2,3-bis[2-methoxy-4-nitro-5-sulfophenyl]-2H-tetrazolium-5-carboxanilide inner salt (XTT)–based assay (Sigma) as described previously [35].

### Cell cycle assay

A propidium iodide-based flow cytometry assay was assessed as described previously [56]. In short, a propidium iodide stain assay is used to segregate cells according to the DNA content, which is graphically shown in a histogram plot (high content in G2/M, intermediate content in S-phase, low content in G1/G0 and lowest content in dead/apoptotic cells, which defines a sub-G1/G0 fraction),

### Data analysis

Linear regression dose effect plots to calculate IC50s were computed with values in between upper and lower threshold doses of minimal/maximal dose effects using Calcusyn Software (Biosoft, Cambridge, UK), which is based on equations provided by Chou and Talaly [37].

Isobologram analyses were performed as we have previously described [54,55]. In short, cells were treated with fixed ratios in relationship to the individual agent ED and data was analyzed using the method of Chou and Talalay to produce isobolograms. This allowed calculation of combination indices (CI). The CI provide a numerical description of the effects of a combination treatment. Specifically, a CI < 1 indicates synergy, a CI = 1 indicates an additive effect, and a CI > 1 indicates antagonism of the two agents.

## Abbreviations

ABL1: Abelson murine leukemia viral oncogene homolog 1; AKT = PKB: Protein kinase B; AML: Acute myeloid leukemia; ALL: Acute lymphoid leukemia; CI: Combination index; CML: Chronic myeloid leukemia; ED50: Effective dose to inhibit 50% of a defined endpoint; EVI1: Ecotropic virus integration site 1; FLT3: FMS-like tyrosine kinase 3; GIST: Gastrointestinal stromal tumor; IC50: Concentration sufficient to achieve a 50% inhibition; IL3: Interleukin 3; ITD: Internal tandem duplication; KIT: v-kit Hardy-Zuckerman 4 feline sarcoma viral oncogene homolog; mTOR: Mammalian target of rapamycin; MTORC1/2: Mammalian target of rapamycin complex 1/2; PI3K: Phosphoinositide 3 kinase; PTEN: Phosphatase and Tensin homolog; Rictor: Rapamycin-insensitive companion of mTOR; Raptor: Regulatory associated protein of mTOR; TK: Tyrosine kinase; TKI: Tyrosine kinase inhibitor; TKD: Tyrosine kinase domain; XTT: 2,3-Bis-(2-methoxy-4-nitro-5-sulfophenyl)-2H-tetrazolium-5-carboxanilid-sodium salt.

## Competing interests

Dr. Kampa-Schittenhelm: no conflicts.

Dr. Heinrich Consultant Novartis, MolecularMD, Research funding: Novartis, Ariad, Imclone, AROG, Equity interest: MolecularMD.

Figen Akmut: no conflicts.

Katharina Henriette Rasp: no conflicts.

Barbara Illing: no conflicts.

Dr. Hartmut Döhner: Consultant: Novartis, Celgene, Boehringer Ingelheim, Ambit.

Dr. Konstanze Döhner: Consultant: Novartis.

Dr. Schittenhelm: no conflicts.

## Authors’ contributions

KS designed research, performed research, analyzed data and wrote the paper. MH analyzed data, and wrote the paper. FA performed research and analyzed data. KR performed research analyzed data. BI performed research analyzed data. HD analyzed data, and wrote the paper. KD analyzed data, and wrote the paper. MS designed research, performed research, analyzed data and wrote the paper. All authors read and approved the final manuscript.

## Supplementary Material

Additional file 1: Table S1 AKT Phospho-Expression Analysis - *Patient Characteristics.* Click here for file

Additional file 2: Figure S1 NVP-BGT226 and NVP-BEZ235 target AKT-mediated viability of native leukemia cells ex vivo. NVP-BGT226 and NVP-BEZ235 target AKT-mediated viability of native leukemia cells. (A) A flow cytometry based assay using native acute leukemia cells treated with NVP-BGT226 or NVP-BEZ235 demonstrates variable proapoptotic efficacy. The average of three acute leukemia patients is shown. Standard deviations reveal relatively high inter-individual differences in sensitivity towards both inhibitors – with NVP-BGT226 being the more potent agent. (B) AKT signaling is a target of dual PI3K/MTOR inhibition in native leukemia blasts. An immunoblot experiment using whole cell lysates of two patients is shown. Actin blotting is used as a loading control. Click here for file

Additional file 3: Figure S2 PI3K/MTOR inhibition in imatinib-resistance models. Dual PI3K/MTOR inhibition is effective in tyrosine kinase inhibitor-resistant cell models. Two cell models, the HMC1 mast cell leukemia cell strains (A and B) and two GIST cell lines (C and D; for more information about the cell lines, see comments below), were established to compare primary imatinib-sensitive versus secondary imatinib-insensitive mutation patterns with regard to sensitivity to NVP-BGT226 (A and C) or NVP-BEZ235 (B and D). Dose-effect plots are provided indicating sensitivity profiles of both dual PI3K/MTOR inhibitors that are independent of the sensitivity patterns for imatinib. Linear regression analyses to calculate IC50 estimates are provided for all cell lines. [HMC1.1: Mast cell leukemia cell line, harboring a *KIT* V560G mutation; HMC1.2: sister cell line of HMC1.1, harboring an additional *KIT* D816V mutation; GIST882: gastrointestinal stromal tumor harboring an imatinib-sensitive *KIT* K642E mutation; GIST48: gastrointestinal stromal tumor harboring an imatinib-sensitive V560D mutation plus a secondary imatinib-insensitive activation loop mutation (D820A)]. Click here for file

## Supported

In part by the Deutsche Krebshilfe Foundation (MMS, KKS), the *f*ortüne-Program of the Tübingen Medical Faculty (Nr. 1490-0-0, MMS and 11836-0-0, KMK), the IZKF Program of the Medical Faculty Tübingen (MMS) and the José Carreras Scholarship Program (KKS), the Ludwig Hiermaier Foundation of the Comprehensive Cancer Center Tübingen (MMS), a Merit Review Grant from the Department of Veterans Affairs (MCH) and a grant from the Leukemia and Lymphoma Society (MCH), GIST Cancer Research Fund (MCH) and the Life Raft Group (MCH).

## References

[B1] Pui CH Relling MV Downing JR Acute lymphoblastic leukemia N Engl J Med 2004 350 1535 1548 10.1056/NEJMra023001 15071128

[B2] Dohner H Estey EH Amadori S Appelbaum FR Buchner T Burnett AK Dombret H Fenaux P Grimwade D Larson RA Diagnosis and management of acute myeloid leukemia in adults: recommendations from an international expert panel, on behalf of the European LeukemiaNet Blood 2010 115 453 474 10.1182/blood-2009-07-235358 19880497

[B3] Druker BJ Sawyers CL Kantarjian H Resta DJ Reese SF Ford JM Capdeville R Talpaz M Activity of a specific inhibitor of the BCR-ABL tyrosine kinase in the blast crisis of chronic myeloid leukemia and acute lymphoblastic leukemia with the Philadelphia chromosome N Engl J Med 2001 344 1038 1042 10.1056/NEJM200104053441402 11287973

[B4] Schittenhelm M Aichele O Krober SM Brummendorf T Kanz L Denzlinger C Complete remission of third recurrence of acute myeloid leukemia after treatment with imatinib (STI-571) Leuk Lymphoma 2003 44 1251 1253 10.1080/1042819031000077034 12916883

[B5] Kindler T Breitenbuecher F Marx A Hess G Gschaidmeier H Gamm H Kirkpatrick CJ Huber C Fischer T Sustained complete hematologic remission after administration of the tyrosine kinase inhibitor imatinib mesylate in a patient with refractory, secondary AML Blood 2003 101 2960 2962 10.1182/blood-2002-05-1469 12480706

[B6] Kindler T Breitenbuecher F Marx A Beck J Hess G Weinkauf B Duyster J Peschel C Kirkpatrick CJ Theobald M Efficacy and safety of imatinib in adult patients with c-kit-positive acute myeloid leukemia Blood 2004 103 3644 3654 10.1182/blood-2003-06-2071 14726395

[B7] Kindler T Lipka DB Fischer T FLT3 as a therapeutic target in AML: still challenging after all these years Blood 2010 116 5089 5102 10.1182/blood-2010-04-261867 20705759

[B8] Manning BD Cantley LC AKT/PKB signaling: navigating downstream Cell 2007 129 1261 1274 10.1016/j.cell.2007.06.009 17604717 PMC2756685

[B9] Kharas MG Okabe R Ganis JJ Gozo M Khandan T Paktinat M Gilliland DG Gritsman K Constitutively active AKT depletes hematopoietic stem cells and induces leukemia in mice Blood 2010 115 1406 1415 10.1182/blood-2009-06-229443 20008787 PMC2826762

[B10] Brandts CH Sargin B Rode M Biermann C Lindtner B Schwable J Buerger H Muller-Tidow C Choudhary C McMahon M Constitutive activation of Akt by Flt3 internal tandem duplications is necessary for increased survival, proliferation, and myeloid transformation Cancer Res 2005 65 9643 9650 10.1158/0008-5472.CAN-05-0422 16266983

[B11] Brognard J Clark AS Ni Y Dennis PA Akt/protein kinase B is constitutively active in non-small cell lung cancer cells and promotes cellular survival and resistance to chemotherapy and radiation Cancer Res 2001 61 3986 3997 11358816

[B12] Vivanco I Sawyers CL The phosphatidylinositol 3-Kinase AKT pathway in human cancer Nat Rev Cancer 2002 2 489 501 10.1038/nrc839 12094235

[B13] Samuels Y Wang Z Bardelli A Silliman N Ptak J Szabo S Yan H Gazdar A Powell SM Riggins GJ High frequency of mutations of the PIK3CA gene in human cancers Science 2004 304 554 10.1126/science.1096502 15016963

[B14] Kim MS Jeong EG Yoo NJ Lee SH Mutational analysis of oncogenic AKT E17K mutation in common solid cancers and acute leukaemias Br J Cancer 2008 98 1533 1535 10.1038/sj.bjc.6604212 18392055 PMC2391109

[B15] Hollander MC Blumenthal GM Dennis PA PTEN loss in the continuum of common cancers, rare syndromes and mouse models Nat Rev Cancer 2011 11 289 301 21430697 10.1038/nrc3037 PMC6946181

[B16] Sos ML Koker M Weir BA Heynck S Rabinovsky R Zander T Seeger JM Weiss J Fischer F Frommolt P PTEN loss contributes to erlotinib resistance in EGFR-mutant lung cancer by activation of Akt and EGFR Cancer Res 2009 69 3256 3261 10.1158/0008-5472.CAN-08-4055 19351834 PMC2849653

[B17] Zenz T Dohner K Denzel T Dohner H Stilgenbauer S Bullinger L Chronic lymphocytic leukaemia and acute myeloid leukaemia are not associated with AKT1 pleckstrin homology domain (E17K) mutations Br J Haematol 2008 141 742 743 10.1111/j.1365-2141.2008.07113.x 18410456

[B18] Tibes R Kornblau SM Qiu Y Mousses SM Robbins C Moses T Carpten JD PI3K/AKT pathway activation in acute myeloid leukaemias is not associated with AKT1 pleckstrin homology domain mutation Br J Haematol 2008 140 344 347 10.1111/j.1365-2141.2007.06920.x 18053070 PMC3385948

[B19] Martelli AM Nyakern M Tabellini G Bortul R Tazzari PL Evangelisti C Cocco L Phosphoinositide 3-kinase/Akt signaling pathway and its therapeutical implications for human acute myeloid leukemia Leukemia 2006 20 911 928 10.1038/sj.leu.2404245 16642045

[B20] Maira SM Finan P Garcia-Echeverria C From the bench to the bed side: PI3K pathway inhibitors in clinical development Curr Top Microbiol Immunol 2010 347 209 239 10.1007/82_2010_60 20582534

[B21] Hudes G Carducci M Tomczak P Dutcher J Figlin R Kapoor A Staroslawska E Sosman J McDermott D Bodrogi I Temsirolimus, interferon alfa, or both for advanced renal-cell carcinoma N Engl J Med 2007 356 2271 2281 10.1056/NEJMoa066838 17538086

[B22] Recher C Beyne-Rauzy O Demur C Chicanne G Dos Santos C Mas VM Benzaquen D Laurent G Huguet F Payrastre B Antileukemic activity of rapamycin in acute myeloid leukemia Blood 2005 105 2527 2534 10.1182/blood-2004-06-2494 15550488

[B23] Schittenhelm M Theil A Shiraga S Lee F Heinrich M Dasatinib and rapamycin synergistically inhibit the proliferation of cells expressing oncogenic KIT kinase via global inhibition of AKT-dependent signaling 2007 AACR Annual Meeting pp. Abstract 5416: AACR Meeting Abstracts; 2007:Abstract 5416

[B24] Tamburini J Chapuis N Bardet V Park S Sujobert P Willems L Ifrah N Dreyfus F Mayeux P Lacombe C Bouscary D Mammalian target of rapamycin (mTOR) inhibition activates phosphatidylinositol 3-kinase/Akt by up-regulating insulin-like growth factor-1 receptor signaling in acute myeloid leukemia: rationale for therapeutic inhibition of both pathways Blood 2008 111 379 382 10.1182/blood-2007-03-080796 17878402

[B25] Carracedo A Ma L Teruya-Feldstein J Rojo F Salmena L Alimonti A Egia A Sasaki AT Thomas G Kozma SC Inhibition of mTORC1 leads to MAPK pathway activation through a PI3K-dependent feedback loop in human cancer J Clin Invest 2008 118 3065 3074 18725988 10.1172/JCI34739 PMC2518073

[B26] O’Reilly KE Rojo F She QB Solit D Mills GB Smith D Lane H Hofmann F Hicklin DJ Ludwig DL mTOR inhibition induces upstream receptor tyrosine kinase signaling and activates Akt Cancer Res 2006 66 1500 1508 10.1158/0008-5472.CAN-05-2925 16452206 PMC3193604

[B27] Chang KY Tsai SY Wu CM Yen CJ Chuang BF Chang JY Novel phosphoinositide 3-kinase/mTOR dual inhibitor, NVP-BGT226, displays potent growth-inhibitory activity against human head and neck cancer cells in vitro and in vivo Clin Cancer Res 2011 17 7116 7126 10.1158/1078-0432.CCR-11-0796 21976531

[B28] Maira SM Stauffer F Brueggen J Furet P Schnell C Fritsch C Brachmann S Chene P De Pover A Schoemaker K Identification and characterization of NVP-BEZ235, a new orally available dual phosphatidylinositol 3-kinase/mammalian target of rapamycin inhibitor with potent in vivo antitumor activity Mol Cancer Ther 2008 7 1851 1863 10.1158/1535-7163.MCT-08-0017 18606717

[B29] Polak R Buitenhuis M The PI3K/PKB signaling module as key regulator of hematopoiesis: implications for therapeutic strategies in leukemia Blood 2012 119 911 923 10.1182/blood-2011-07-366203 22065598

[B30] Gallay N Dos Santos C Cuzin L Bousquet M Simmonet Gouy V Chaussade C Attal M Payrastre B Demur C Recher C The level of AKT phosphorylation on threonine 308 but not on serine 473 is associated with high-risk cytogenetics and predicts poor overall survival in acute myeloid leukaemia Leukemia 2009 23 1029 1038 10.1038/leu.2008.395 19158829

[B31] Shan X Czar MJ Bunnell SC Liu P Liu Y Schwartzberg PL Wange RL Deficiency of PTEN in Jurkat T cells causes constitutive localization of Itk to the plasma membrane and hyperresponsiveness to CD3 stimulation Mol Cell Biol 2000 20 6945 6957 10.1128/MCB.20.18.6945-6957.2000 10958690 PMC88770

[B32] Chapuis N Tamburini J Green AS Vignon C Bardet V Neyret A Pannetier M Willems L Park S Macone A Dual inhibition of PI3K and mTORC1/2 signaling by NVP-BEZ235 as a new therapeutic strategy for acute myeloid leukemia Clin Cancer Res 2010 16 5424 5435 10.1158/1078-0432.CCR-10-1102 20884625

[B33] Panwalkar A Verstovsek S Giles FJ Mammalian target of rapamycin inhibition as therapy for hematologic malignancies Cancer 2004 100 657 666 10.1002/cncr.20026 14770419

[B34] Bach M Larance M James DE Ramm G The serine/threonine kinase ULK1 is a target of multiple phosphorylation events Biochem J 2011 440 283 291 10.1042/BJ20101894 21819378

[B35] Schittenhelm MM Shiraga S Schroeder A Corbin AS Griffith D Lee FY Bokemeyer C Deininger MW Druker BJ Heinrich MC Dasatinib (BMS-354825), a dual SRC/ABL kinase inhibitor, inhibits the kinase activity of wild-type, juxtamembrane, and activation loop mutant KIT isoforms associated with human malignancies Cancer Res 2006 66 473 481 10.1158/0008-5472.CAN-05-2050 16397263

[B36] Schittenhelm MM Yee KW Tyner JW McGreevey L Haley AD Town A Griffith DJ Bainbridge T Braziel RM O'Farrell AM FLT3 K663Q is a novel AML-associated oncogenic kinase: Determination of biochemical properties and sensitivity to Sunitinib (SU11248) Leukemia 2006 20 2008 2014 10.1038/sj.leu.2404374 16990784

[B37] Chou TC Talalay P Quantitative analysis of dose-effect relationships: the combined effects of multiple drugs or enzyme inhibitors Adv Enzyme Regul 1984 22 27 55 6382953 10.1016/0065-2571(84)90007-4

[B38] Kirstein MM Boukouris AE Pothiraju D Buitrago-Molina LE Marhenke S Schutt J Orlik J Kuhnel F Hegermann J Manns MP Vogel A Activity of the mTOR inhibitor RAD001, the dual mTOR and PI3-kinase inhibitor BEZ235 and the PI3-kinase inhibitor BKM120 in hepatocellular carcinoma Liver Int 2013 10.1111/liv.12126 23489999

[B39] Baumann P Schneider L Mandl-Weber S Oduncu F Schmidmaier R Simultaneous targeting of PI3K and mTOR with NVP-BGT226 is highly effective in multiple myeloma Anticancer Drugs 2012 23 131 138 10.1097/CAD.0b013e32834c8683 21959532

[B40] Smith CC Wang Q Chin CS Salerno S Damon LE Levis MJ Perl AE Travers KJ Wang S Hunt JP Validation of ITD mutations in FLT3 as a therapeutic target in human acute myeloid leukaemia Nature 2012 485 260 263 10.1038/nature11016 22504184 PMC3390926

[B41] Bauer S Duensing A Demetri GD Fletcher JA KIT oncogenic signaling mechanisms in imatinib-resistant gastrointestinal stromal tumor: PI3-kinase/AKT is a crucial survival pathway Oncogene 2007 26 7560 7568 10.1038/sj.onc.1210558 17546049

[B42] Furitsu T Tsujimura T Tono T Ikeda H Kitayama H Koshimizu U Sugahara H Butterfield JH Ashman LK Kanayama Y Identification of mutations in the coding sequence of the proto-oncogene c-kit in a human mast cell leukemia cell line causing ligand-independent activation of c-kit product J Clin Invest 1993 92 1736 1744 10.1172/JCI116761 7691885 PMC288334

[B43] Ma Y Zeng S Metcalfe DD Akin C Dimitrijevic S Butterfield JH McMahon G Longley BJ The c-KIT mutation causing human mastocytosis is resistant to STI571 and other KIT kinase inhibitors; kinases with enzymatic site mutations show different inhibitor sensitivity profiles than wild-type kinases and those with regulatory-type mutations Blood 2002 99 1741 1744 10.1182/blood.V99.5.1741 11861291

[B44] Tuveson DA Willis NA Jacks T Griffin JD Singer S Fletcher CD Fletcher JA Demetri GD STI571 inactivation of the gastrointestinal stromal tumor c-KIT oncoprotein: biological and clinical implications Oncogene 2001 20 5054 5058 10.1038/sj.onc.1204704 11526490

[B45] Bauer S Yu LK Demetri GD Fletcher JA Heat shock protein 90 inhibition in imatinib-resistant gastrointestinal stromal tumor Cancer Res 2006 66 9153 9161 10.1158/0008-5472.CAN-06-0165 16982758

[B46] Breitenbuecher F Markova B Kasper S Carius B Stauder T Bohmer FD Masson K Ronnstrand L Huber C Kindler T Fischer T A novel molecular mechanism of primary resistance to FLT3-kinase inhibitors in AML Blood 2009 113 4063 4073 10.1182/blood-2007-11-126664 19144992

[B47] Groschel S Lugthart S Schlenk RF Valk PJ Eiwen K Goudswaard C van Putten WJ Kayser S Verdonck LF Lubbert M High EVI1 expression predicts outcome in younger adult patients with acute myeloid leukemia and is associated with distinct cytogenetic abnormalities J Clin Oncol 2010 28 2101 2107 10.1200/JCO.2009.26.0646 20308656

[B48] Yoshimi A Goyama S Watanabe-Okochi N Yoshiki Y Nannya Y Nitta E Arai S Sato T Shimabe M Nakagawa M Evi1 represses PTEN expression and activates PI3K/AKT/mTOR via interactions with polycomb proteins Blood 2011 117 3617 3628 10.1182/blood-2009-12-261602 21289308

[B49] Burris H Rodon J Sharma S Herbst R Tabernero J Infante J Silva A Demanse D Hackl H Baselga J First-in-human phase I study of the oral PI3K inhibitor BEZ235 in patients (pts) with advanced solid tumors. In ASCO Annual Meeting J Clin Oncol 2010 28 Abstract 3005

[B50] Markman B Tabernero J Krop I Shapiro GI Siu L Chen LC Mita M Melendez Cuero M Stutvoet S Birle D Phase I safety, pharmacokinetic, and pharmacodynamic study of the oral phosphatidylinositol-3-kinase and mTOR inhibitor BGT226 in patients with advanced solid tumors Ann Oncol 2012 23 2399 2408 10.1093/annonc/mds011 22357447

[B51] Glienke W Maute L Wicht J Bergmann L The dual PI3K/mTOR inhibitor NVP-BGT226 induces cell cycle arrest and regulates Survivin gene expression in human pancreatic cancer cell lines Tumour Biol 2012 33 757 765 10.1007/s13277-011-0290-2 22170433

[B52] Quentmeier H Reinhardt J Zaborski M Drexler HG FLT3 mutations in acute myeloid leukemia cell lines Leukemia 2003 17 120 124 10.1038/sj.leu.2402740 12529668

[B53] Frohling S Scholl C Levine RL Loriaux M Boggon TJ Bernard OA Berger R Dohner H Dohner K Ebert BL Identification of driver and passenger mutations of FLT3 by high-throughput DNA sequence analysis and functional assessment of candidate alleles Cancer Cell 2007 12 501 513 10.1016/j.ccr.2007.11.005 18068628

[B54] Yee KW Schittenhelm M O'Farrell AM Town AR McGreevey L Bainbridge T Cherrington JM Heinrich MC Synergistic effect of SU11248 with cytarabine or daunorubicin on FLT3 ITD-positive leukemic cells Blood 2004 104 4202 4209 10.1182/blood-2003-10-3381 15304385

[B55] Schittenhelm MM Kampa KM Yee KW Heinrich MC The FLT3 inhibitor tandutinib (formerly MLN518) has sequence-independent synergistic effects with cytarabine and daunorubicin Cell Cycle 2009 8 2621 2630 10.4161/cc.8.16.9355 19625780

[B56] Mueller S Schittenhelm M Honecker F Malenke E Lauber K Wesselborg S Hartmann JT Bokemeyer C Mayer F Cell-cycle progression and response of germ cell tumors to cisplatin in vitro Int J Oncol 2006 29 471 479 16820891

