# Supplementary material for: Cell cycle-dependent activity of the novel dual PI3K-MTORC1/2 inhibitor NVP-BGT226 in acute leukemia
Source: Mol Cancer. 2013 May 24;12:46. doi: 10.1186/1476-4598-12-46 (PMC3689638; doi:10.1186/1476-4598-12-46)

**Table S1.**

**AKT Phospho-Expression Analysis - *Patient Characteristics***


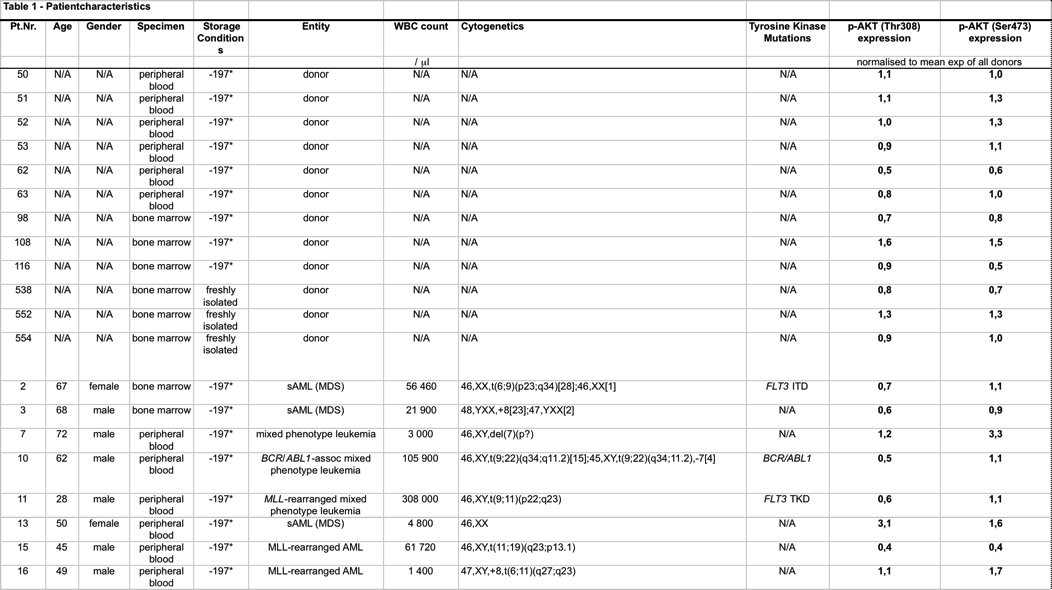

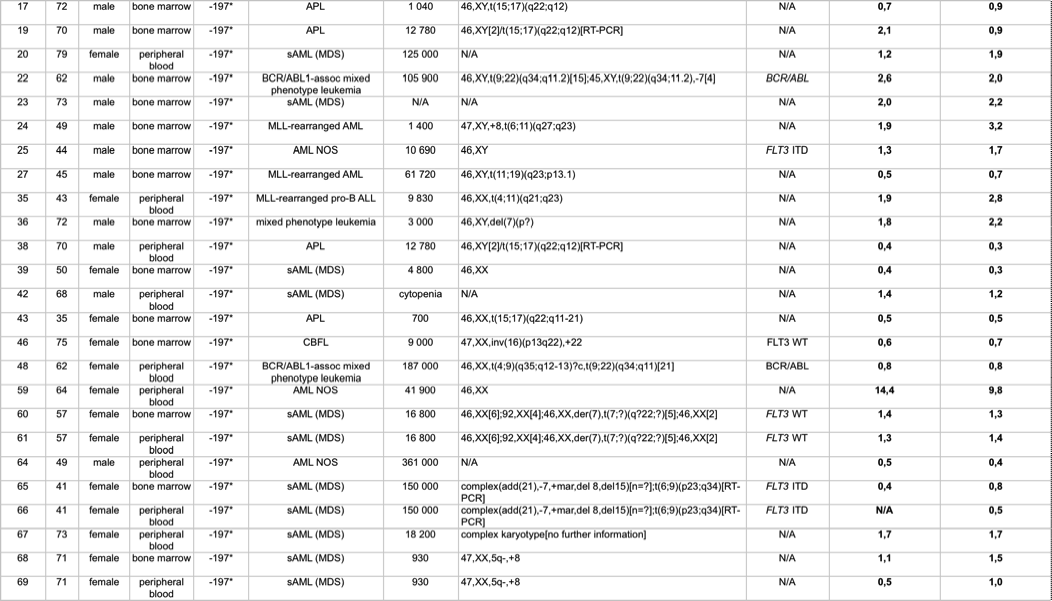


***Footnotes:*** *N/A: not available; AML NOS: acute myeloid leukemia not otherwise specified; sAML (MDS): secondary AML derived from myelodysplastic syndrome; APL: acute promyelocytic leukemia; CBFL: core binding factor leukemia; tAML: therapy-related AML; mut-NPM1 AML: AML with mutated NPM1*


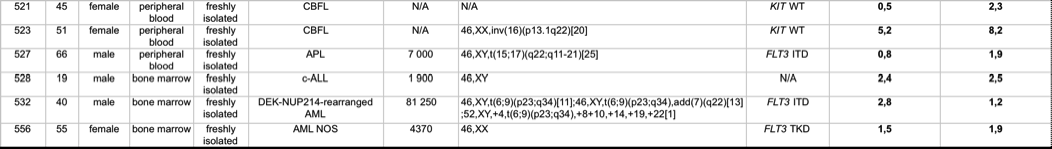


*(provisional entity); BCR/ABL1-assoc leukemia: leukemia with t(9;22)(q34;q11.2); MLL-assoc. leukemia: leukemia with t(v;11q23); c-ALL: common acute lymphoblastic leukemia; WT: wildtype isoform; ITD: internal tandem duplication mutation; TKD: autoactivating mutation located in the tyrosine kinase domain.*


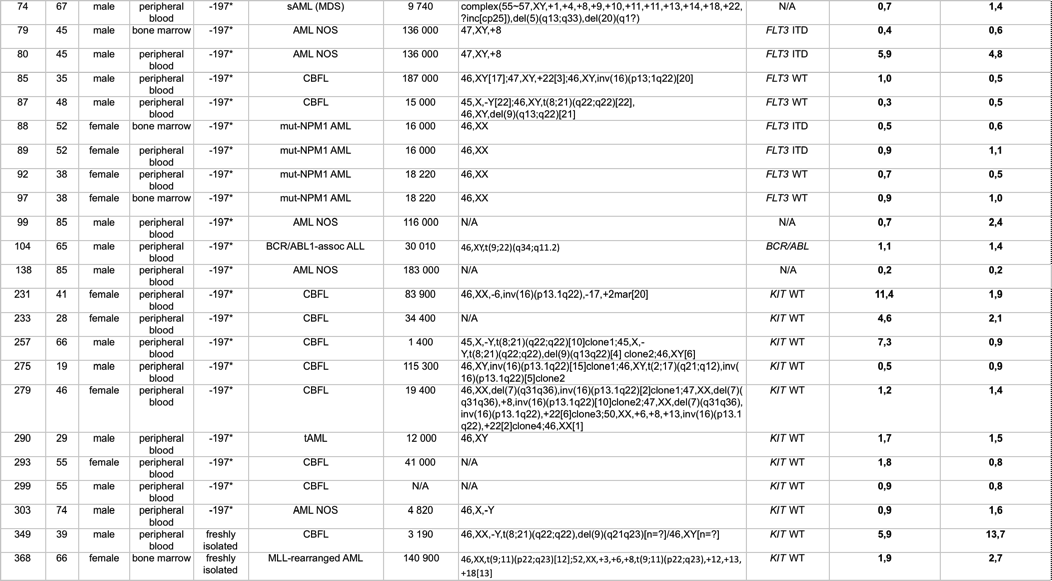

Supplement: Additional file 1: Table S1 — AKT Phospho-Expression Analysis - Patient Characteristics. [file 1476-4598-12-46-S1.docx]
