# Supplementary figures and images for: Cell cycle-dependent activity of the novel dual PI3K-MTORC1/2 inhibitor NVP-BGT226 in acute leukemia
Source: Mol Cancer. 2013 May 24;12:46. doi: 10.1186/1476-4598-12-46 (PMC3689638; doi:10.1186/1476-4598-12-46)

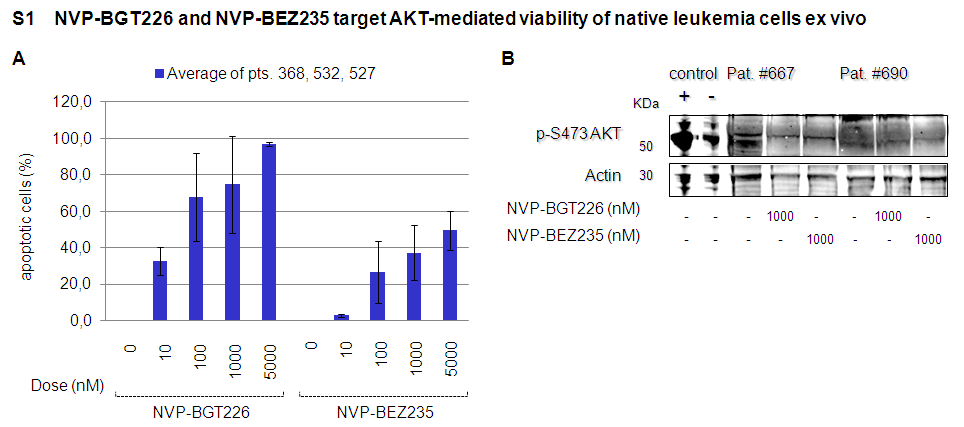

Supplement: Additional file 2: Figure S1 — NVP-BGT226 and NVP-BEZ235 target AKT-mediated viability of native leukemia cells ex vivo. NVP-BGT226 and NVP-BEZ235 target AKT-mediated viability of native leukemia cells. (A) A flow cytometry based assay using native acute leukemia cells treated with NVP-BGT226 or NVP-BEZ235 demonstrates variable proapoptotic efficacy. The average of three acute leukemia patients is shown. Standard deviations reveal relatively high inter-individual differences in sensitivity towards both inhibitors – with NVP-BGT226 being the more potent agent. (B) AKT signaling is a target of dual PI3K/MTOR inhibition in native leukemia blasts. An immunoblot experiment using whole cell lysates of two patients is shown. Actin blotting is used as a loading control. [file 1476-4598-12-46-S2.tiff]

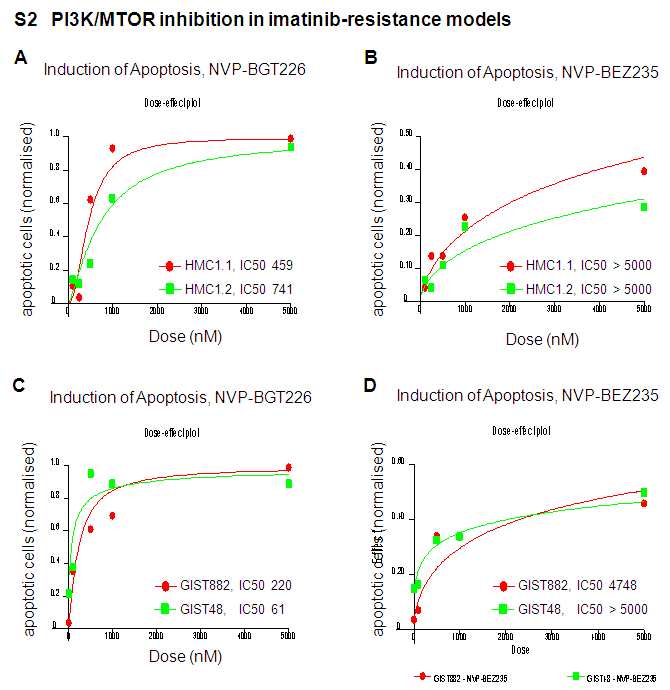

Supplement: Additional file 3: Figure S2 — PI3K/MTOR inhibition in imatinib-resistance models. Dual PI3K/MTOR inhibition is effective in tyrosine kinase inhibitor-resistant cell models. Two cell models, the HMC1 mast cell leukemia cell strains (A and B) and two GIST cell lines (C and D; for more information about the cell lines, see comments below), were established to compare primary imatinib-sensitive versus secondary imatinib-insensitive mutation patterns with regard to sensitivity to NVP-BGT226 (A and C) or NVP-BEZ235 (B and D). Dose-effect plots are provided indicating sensitivity profiles of both dual PI3K/MTOR inhibitors that are independent of the sensitivity patterns for imatinib. Linear regression analyses to calculate IC50 estimates are provided for all cell lines. [HMC1.1: Mast cell leukemia cell line, harboring a KIT V560G mutation; HMC1.2: sister cell line of HMC1.1, harboring an additional KIT D816V mutation; GIST882: gastrointestinal stromal tumor harboring an imatinib-sensitive KIT K642E mutation; GIST48: gastrointestinal stromal tumor harboring an imatinib-sensitive V560D mutation plus a secondary imatinib-insensitive activation loop mutation (D820A)]. [file 1476-4598-12-46-S3.tiff]
